# PSAT1 Promotes NSCLC Progression via the *De Novo* Serine Synthesis Pathway and Represents a Therapeutic Vulnerability

**DOI:** 10.1002/cam4.71780

**Published:** 2026-04-02

**Authors:** Xijia Zhou, Min Zhao, Yingshu Cao, Xiangyu Zhou, Ke Wang

**Affiliations:** ^1^ Department of Respiratory and Critical Care Medicine the Second Hospital of Jilin University Changchun China

**Keywords:** apoptosis, *de novo* serine synthesis pathway, non‐small cell lung cancer, PSAT1, ROS

## Abstract

**Background:**

Non‐small cell lung cancer (NSCLC) is a malignant tumor characterized by high morbidity and mortality, as well as metabolic reprogramming. Enhanced serine synthesis plays a crucial role in the aberrant metabolism of NSCLC. Among the three key enzymes involved in serine synthesis, phosphoserine aminotransferase 1 (*PSAT1*) requires further investigation to elucidate its regulatory mechanisms in NSCLC.

**Methods:**

In this study, we employed bioinformatics analysis, immunohistochemistry, CCK‐8 assay, colony formation assay, flow cytometry assay, isotope tracing technique, WB analysis, and nude mouse xenograft models to validate the expression and function of *PSAT1* in NSCLC.

**Results:**

Our results demonstrated that *PSAT1* was significantly upregulated in NSCLC cells and contributed to promoting cell proliferation, inhibiting apoptosis, and attenuating the efficacy of gefitinib treatment. Moreover, knockdown of *PSAT1* led to inhibition of the *de novo* serine synthesis pathway (SSP), elevation of reactive oxygen species (ROS) levels, and activation of the mitochondrial apoptotic pathway. Notably, combined knockdown of *PSAT1* with exogenous serine intake inhibition synergistically suppressed NSCLC progression.

**Conclusion:**

Collectively, our findings highlight that *PSAT1* serves as a biomarker for metabolic reprogramming in NSCLC and exhibits a close association with disease development and treatment.

**Abbreviations**
1C: one‐carbon
3‐PG: 3‐phosphoglycerate
GSH: Reduced glutathione
GSSG: Oxidized glutathione disulfide
IHC: Immunohistochemistry
NADP: Nicotinamide adenine dinucleotide phosphate
NADPH: Nicotinamide adenine dinucleotide phosphate hydrogen
NSCLC: Non‐small cell lung cancer
*PHGDH*
: 3‐phosphoglycerate dehydrogenase
*PSAT1*
: Phosphoserine aminotransferase 1
*PSPH*
: phosphoserine phosphatase
ROS: Reactive oxygen species
SSP: *de novo* serine synthesis pathway

## 1 Introduction

Lung cancer, the leading cause of cancer‐related mortality [1], is classified into two histological subtypes: Non‐small‐cell lung cancer (NSCLC; 82%) and small‐cell lung cancer (SCLC; 14%), with approximately 3% of cases having unspecified histology [2]. Due to late diagnosis, chemotherapy resistance, and metastasis, the five‐year survival rate for NSCLC patients remains poor [3, 4, 5]. Therefore, it is imperative to discover novel therapeutic targets for NSCLC.

Metabolic reprogramming is a hallmark of malignant tumors, and the “Warburg effect” is a well‐established mode of metabolic reprogramming [6, 7]. The “Warburg effect” refers to the phenomenon in which normal differentiated cells primarily rely on mitochondrial oxidative phosphorylation for energy supply, while most tumor cells depend on aerobic glycolysis [8]. One explanation for this phenomenon is that the “Warburg effect” represents a “selfish” metabolic reprogramming of tumor cells, promoting biosynthesis of nucleotides, non‐essential amino acids, and other molecules through diversion of glycolytic intermediates [9]. In the *de novo* serine synthesis pathway (SSP), 3‐phosphoglycerate (3‐PG), an intermediate product of glycolytic metabolism, is catalyzed by 3‐phosphoglycerate dehydrogenase (*PHGDH*), phosphoserine aminotransferase 1 (*PSAT1*), and phosphoserine phosphatase (*PSPH*) to produce serine [10]. SSP integrates with folate and one‐carbon (1C) metabolism, regulating nucleotide synthesis, methylation, and redox balance [11, 12], making it critical for cancer proliferation.

Tumor cells undergo metabolic reprogramming to support cell proliferation and metastasis [13], resulting in tumor cell dependencies and vulnerabilities for targeted therapy [14]. Serine, a non‐essential amino acid, can be obtained through dietary intake or synthesized via SSP. Serine serves as a foundation for nucleotide synthesis, nicotinamide adenine dinucleotide phosphate hydrogen (NADPH) production, and glutathione formation [15]. Studies have demonstrated that reducing exogenous serine intake and inhibiting endogenous serine production can inhibit tumor progression [16]. For example, arginine methylation enhances the catalytic activity of *PHGDH*, promoting endogenous serine synthesis and indicating a vulnerability in hepatocellular carcinoma [17]. Additionally, research has shown that restricting serine availability leads to the accumulation of toxic sphingolipids that inhibit tumor cells both in vivo and in vitro [18]. Research on serine metabolism is thus expected to provide new directions for the metabolic treatment of tumors.

*PSAT1* is the second key enzyme in SSP and catalyzes the deamination of glutamate to α‐ketoglutaric acid (α‐KG) [10, 19]. A proteogenomic landscape study involving 108 cases of squamous cell lung cancer revealed a significant increase in *PSAT1* expression in lung cancer tissues, suggesting its potential vulnerability as a therapeutic target for squamous cell lung cancer [20]. One study demonstrated that elevated levels of *PSAT1* inhibit cyclin D1 degradation, thereby promoting the progression and proliferation of NSCLC [21]. Another study suggested that simultaneously inhibiting both *PSAT1* and exogenous serine intake can induce metabolic disorders in colon cancer and maximize the inhibition of cancer cell proliferation [22]. Furthermore, *PSAT1* has been identified as a diagnostic and prognostic marker for ovarian cancer and may serve as a potential target for treatment [23]. Further exploration into the role of *PSAT1* in metabolic abnormalities associated with NSCLC is necessary to establish a solid foundation for the treatment of NSCLC. Given that serine metabolism is pivotal for glutathione synthesis and redox homeostasis [24], and that elevated reactive oxygen species (ROS) are known potent activators of the Nuclear Factor‐kappa B (NF‐κB) pathway [25], we postulated that PSAT1, by governing serine flux, might influence NSCLC progression through ROS‐mediated NF‐κB activation. In this study, we hypothesized that the abnormally high expression of *PSAT1* plays an important role in influencing aberrant serine metabolism in NSCLC and represents a targetable vulnerability. We conducted both in vivo and in vitro experiments to demonstrate the regulatory effect of *PSAT1* on NSCLC cell metabolism.

## 2 Materials and Methods

### 2.1 Bioinformatics Analysis

Gene Expression Profiling Interactive Analysis (GEPIA, http://gepia.cancer‐pku.cn/) was used to determine the differential expression of *PSAT1* in NSCLC and normal lung tissues. The Kaplan–Meier Plotter (http://kmplot.com/analysis/, 2023.09.13 updates) was used to analyze the impact of high *PSAT1* expression on the overall survival (OS) of patients with NSCLC to reflect the predictive effect of high *PSAT1* expression on patient prognosis.

### 2.2 Sample Collection and Immunohistochemistry

The research protocol was reviewed and approved by the Ethics Committee of the Second Hospital of Jilin University under the reference number (2023) Yan Shen No. (194). We analyzed tumor tissues from 15 primary NSCLC patients (surgery: December 2018–February 2022) and a tissue microarray (HLug‐NSCLC150PT‐01, Outdo Biotech, Shanghai, China) containing 75 NSCLC and 75 paired adjacent non‐cancerous tissues. For immunohistochemistry (IHC), paraffin‐embedded sections were dewaxed, hydrated, antigen‐retrieved, and blocked (3% H_2_O_2_ and 10% goat serum), then incubated with PSAT1 antibody (ab232944, Abcam; 1:150) followed by secondary antibody (37°C, 15 min) and DAB/hematoxylin staining. Staining intensity was divided into four levels (0, none; 1, weak; 2, moderate; and 3, strong) according to the color depth (brown; Figure S1). Distribution was classified according to the extent of staining (area covered) and scored as 1 (≤ 25%), 2 (26%–50%), 3 (51%–75%), or 4 (76%–100%). The final result was based on the product of the two scores. A score ≤ 7 indicated a low expression group and a score > 7 a high expression group.

### 2.3 Cell Culture and Transfection

The human NSCLC cell lines A549 (Chinese Academy of Medical Sciences, Beijing, China), H1299, and PC‐9 (both from Procell Life Science&Technology Co. Ltd. Wuhan, China) were cultured in Ham's F‐12 K (Kaighn's) (Gibco, Carlsbad, CA, USA), RPMI 1640 (Gibco), or DMEM (Gibco) media, respectively, each supplemented with 10% fetal bovine serum (FBS; Hyclone, Logan, UT, USA). Customized serine‐deficient culture medium (‐SL) (BOSTER, Wuhan, China) was used to simulate conditions of exogenous serine deficiency. The cells were divided into four groups: Control and normal culture medium (NC‐NM), control and Serine‐lacking culture medium (NC‐SL), *PSAT1* knockdown and normal culture medium (shA‐NM), and *PSAT1* knockdown and serine‐lacking culture medium (shA‐SL). The cells were all incubated at 37°C with 5% CO2 in an incubator. The cells were identified by the short tandem repeat (STR) method, and all cells were free from contamination, such as with bacteria or mycoplasma.

For cell transfection, we constructed three shRNAs using the lentiviral plasmids psi‐LVRU6GP and psi‐LVRU6P as vectors to knock down the expression of *PSAT1*. NSCLC cells were seeded in 6‐well plates and transfected when cell fusion reached 50%. Transfection was performed using Lipofectamine 3000 (Thermo Fisher Scientific, USA) according to the manufacturer's protocol. After transfection, puromycin was used to screen successfully transfected NSCLC cells.

### 2.4 Quantitative Real‐Time PCR (RT‐qPCR)

The transfected cells were collected into ribonuclease/deoxyribonuclease‐free tubes and fully lysed using TransZol Up (TransGen Biotech, Beijing, China). A PrimeScript RT reagent Kit with gDNA Eraser (RR047A, Takara, Japan) was used to reverse transcribe total RNA into complementary DNA (cDNA). The TB Green Premix Ex Taq II Kit (RR820A, Takara, Japan) was then used to perform RT‐qPCR on a LightCycler480 (Roche, Switzerland) system. The RT‐qPCR conditions were 95°C for 30 s, 40 cycles at 95°C for 10 s, and 60°C for 60 s.

The primers designed for RT‐qPCR analysis were as follows:

*PSAT1* forward: 5′ TGCCGCACTCAGTGTTGTTAG 3′.

*PSAT1* reverse: 5′ GCAATTCCCGCACAAGATTCT3′.

*β‐Actin* forward: 5′ GTGGCCGAGGACTTTGATTG 3′.

*β‐Actin* reverse: 5′ CCTGTAACAACGCATCTCATATT 3′.

### 2.5 Western Blotting (WB) and Antibodies

Cells were lysed on ice using RIPA buffer supplemented with protease and phosphatase inhibitors. The supernatants were collected after centrifugation, and protein concentration was determined using the BCA concentration detection kit (Beyotime, Jiangsu, China). The WB experiments were conducted according to standard operating procedures. The antibodies and dilution ratios used were as follows: Anti‐PSAT1 (10,501–1‐AP, at 1:5000 dilution, Proteintech), anti‐β‐Tubulin (10,094–1‐AP, at 1:2000 dilution, Proteintech), anti‐phosphorylated NF‐κB (p‐NF‐κB) (AP1294, at 1:1000 dilution, ABclonal), anti‐NF‐κB (10,745–1‐AP, at 1:1000 dilution, Proteintech), anti‐Cysteine‐dependent Aspartate‐specific Protease 3 (Caspase 3) (A19654, at 1:1000 dilution, ABclonal), anti‐B‐cell lymphoma‐2 (Bcl‐2) (A19693, at 1:1000 dilution, ABclonal), anti‐Bcl‐2‐Associated X protein (Bax) (A20227, at 1:1000 dilution, ABclonal), anti‐Cytochrome C (A4912, at 1:1000 dilution, ABclonal). The original images of all blots are shown in the Figures S1–S8.

### 2.6 Cell Viability Assay

To determine gefitinib sensitivity, attached cells were treated with a gradient of gefitinib concentrations overnight prior to the assay. For cell proliferation assessment, diluted CCK‐8 solution (CCK‐8: phosphate‐buffered saline [PBS] = 1:9) was added after 24 and 48 h of cell growth, and the absorbance of each well at 450 nm was measured after 1 h of incubation. The same CCK‐8 protocol was followed for the gefitinib‐treated cells.

For the colony formation assay, transfected cells were seeded into 6‐well plates at a density of 1000 cells per well. Complete culture medium was used to culture cells for 5–7 days until cell clones were formed. We removed the culture medium, fixed the cells with methanol, stained them with crystal violet, and observed the formation of cell clones under a microscope (Olympus, Tokyo, Japan). We compared differences in the colony formation ability of the cells in each group based on the crystal violet staining area.

For the Calcein/Propidium Iodide (PI) cell viability/cytotoxicity assay, transfected cells were inoculated onto 14 mm diameter cell slides in a 24‐well plate and cultured in RPMI 1640 medium and serine‐deficient medium for 24 h. The Calcein AM/PI detection working solution was configured according to the instructions in the Calcein/PI Cell Viability/Cytotoxicity Assay Kit (C2015S, Beyotime) and added to the cells at a volume of 250 μL per well. Staining was observed under a fluorescence microscope after 30 min. Green and red fluorescence represent living and dead cells, respectively.

### 2.7 Cell Apoptosis Assay

Transfected cells were collected into centrifuge tubes and detected using the FITC Annexin V Apoptosis Detection Kit (556,547, BD Biosciences, USA). For the assay, 100 μL of binding buffer, 5 μL of FITC Annexin V, and 5 μL of PI were added to every 1 × 10^6^ cells. The cells were gently vortexed and incubated for 15 min at 25°C in the dark. Finally, 400 μL of binding buffer was added to the cells, followed by analysis on a BD FACSCanto II within 60 min. The total cell percentages in Q2 and Q4 quadrants represent apoptotic cells.

### 2.8 Flow Cytometry for Active Caspase‐3

Activation of caspase‐3 was assessed using the Cleaved Caspase‐3 Staining Kit (FITC) (ab65613, Abcam). Transfected cells were collected and stained with FITC‐DEVD‐FMK according to the manufacturer's instructions. The cells were incubated for 60 min at 37°C in the dark. After incubation, cells were centrifuged, the supernatant was removed, and the cells were washed twice with the provided Wash Buffer. Finally, cells were resuspended in Wash Buffer IV and analyzed immediately on a BD FACSCanto II. Cleaved Caspase‐3‐positive cells were quantified to indicate apoptosis.

### 2.9 Flow Cytometry for Cell‐Cycle Phase Detection

Transfected cells were collected into centrifuge tubes and detected using PI/RNase Staining Buffer (550,825, BD Biosciences). Next, 5 mL of 75% ethanol was added to each tube to fix the cells for more than 18 h in the dark. The cells were washed with PBS and stain buffer (FBS) (554,656, BD Biosciences). Then, 0.5 mL of PI/RNase staining buffer was added to every 1 × 10^6^ cells, followed by incubation in the dark for 15 min, and cell‐cycle phase detection on a BD FACSCanto II.

### 2.10 Intracellular ROS Production Assays

For the ROS production assay, treated cells were inoculated onto 14 mm diameter cell slides in a 24‐well plate and detected using the Reactive Oxygen Species Assay Kit (S0033S, Beyotime). We removed the cell culture medium, added 200 μL of diluted DCFH‐DA (DCFH‐DA: RPMI 1640 = 1:1000) to each well, and cultivated the cells in a 37°C incubator for 20 min. The cells were washed three times with serum‐free medium, and DAPI staining solution (C1005, Beyotime) was added for 5 min. We used a fluorescence microscope to observe cell staining and capture photographs. Blue fluorescence represents the cell nucleus, and red fluorescence signal intensity represents the level of ROS.

Glutathione is a small peptide consisting of three amino acid residues and exists in two forms: Oxidized glutathione disulfide (GSSG) and reduced glutathione (GSH). The GSH/GSSG ratio was determined using the GSH and GSSG Assay Kit (S0053, Beyotime). The working solution was prepared according to the instructions, and standard curves were generated. Treated cells were lysed using liquid nitrogen and a 37°C water bath for detecting total glutathione and GSSG. The formula for calculating GSH content is as follows: GSH = Total glutathione—GSSG × 2.

Nicotinamide adenine dinucleotide phosphate (NADP) is a coenzyme involved in many redox reactions and has two forms: NADP+ and NADPH. The NADPH/NADP+ ratio was determined using an NADP+/NADPH Assay Kit with WST‐8 (S0179, Beyotime). We prepared the working solution according to the instructions and generated standard curves. Treated cells were lysed using the NADP+/NADPH extract solution and detected using the G6PDH working solution.

### 2.11 NF‐κB Pathway Inhibition Assay

To investigate the functional role of NF‐κB activation in *PSAT1*‐knockdown cells under serine deprivation, the specific NF‐κB inhibitor BAY 11–7082 (HY‐13453, MCE, USA) was employed. Based on preliminary experiments, an optimal concentration of 10 μM and an incubation period of 24 h were selected. Intracellular ROS levels were quantified using the Reactive Oxygen Species Assay Kit (S0033S, Beyotime) with fluorescence measured at Ex/Em = 488/525 nm via a microplate reader. Apoptosis was assessed in parallel using flow cytometry with the PI/RNase Staining Buffer (550,825, BD Biosciences), as described above.

### 2.12 Isotope Tracer Experiment

Transfected cells were seeded in 6‐well plates at 20% confluence. The tracer culture medium was prepared at normal proportions using U‐^13^C‐labeled glucose (tracer) and RPMI 1640 culture medium without glucose (BOSTER, Wuhan, China). The tracer medium was filtered using a 0.22 μm filter membrane, and dialyzed FBS (Metabo‐Profile Biotechnology Co. Ltd. Shanghai, China) was added, accounting for 10% of the total volume. Three biological replicates and one blank were included per group. Metabolites were extracted and stored at −80°C. We used the UPLC‐TQ‐MS liquid chromatography platform from Metabo‐Profile Biotechnology Co. Ltd. to detect isotope‐labeled metabolites in our biological samples. The project was performed under the guidance of a quality management system. Before the detection, we also set up a blank reagent group of high‐purity reagents to remove the matrix effect accumulated on the chromatographic column. We used MassLynx software (v4.1, Waters, Milford, MA, USA) for peak extraction, integration, identification, and quantitative analysis of each metabolite. When processing experimental data, we only counted the percentage of fully labeled metabolites (such as M + 3 serine) in the total metabolites, and did not count incompletely labeled metabolites (such as M + 2 serine). The statistical data has been processed in advance by deducting blank responses and the blank mass distribution vector (MDV).

### 2.13 Xenograft Model in Nude Mice

The 5‐week‐old female specific pathogen‐free (SPF) grade BALB/c‐nude mice were purchased from SPF (Beijing) Biotechnology Co. Ltd. Twenty nude mice were randomly divided into four groups: Injection of negative control cells and normal diet (NC‐NM), or serine‐deficient feed (NC‐SL), and injection of experimental cells and normal diet (shA‐NM), or serine‐deficient feed (shA‐SL). Transfected H1299 cells were harvested and resuspended in PBS at a density of 2 million cells per 100 μL of liquid. To construct the xenograft model, we injected 2 million cells into each mouse. Fourteen days after cell injection, we observed that tumors had begun to form. The diet of the SL group mice was replaced with a serine‐deficient diet to simulate the lack of serine intake. On day 45, we euthanized the mice by inhalation of a high concentration of isoflurane and removed the tumor tissues for staining experiments.

### 2.14 Statistical Analysis

Data are presented as mean ± SD. Sample size (n) denotes independent biological replicates (CCK‐8: *n* = 6; other in vitro assays: *n* = 3), mice per group (*n* = 5), or patient samples (NSCLC = 84, para‐cancer = 86). Normality was assumed for continuous data. Outliers (Z‐score > 3) were excluded. Western blot and RT‐qPCR data were normalized to β‐tubulin or β‐Actin.

For in vitro/in vivo factorial experiments (factors: *PSAT1* expression and serine availability), planned comparisons between specific groups were analyzed by unpaired two‐tailed Student's *t*‐tests (NC‐SL vs. NC‐NM; shA‐NM vs. NC‐NM; shA‐SL vs. NC‐SL and shA‐SL vs. shA‐NM). The other two‐group comparisons used *t*‐tests. Clinicopathological correlations used the Chi‐square test. Survival differences were assessed by the log‐rank test. Gefitinib IC_50_ was determined by nonlinear regression.

A two‐sided *p* < 0.05 was considered significant (*p < 0.05, **p < 0.01, ***p < 0.001, ****p < 0.0001). Analyses used GraphPad Prism 8.3, IBM SPSS 25.0, ImageJ, and Modfit LT.

## 3 Results

### 3.1 PSAT1 Is Highly‐Expressed in NSCLC and Associated With Poor Prognosis

**FIGURE 1:**
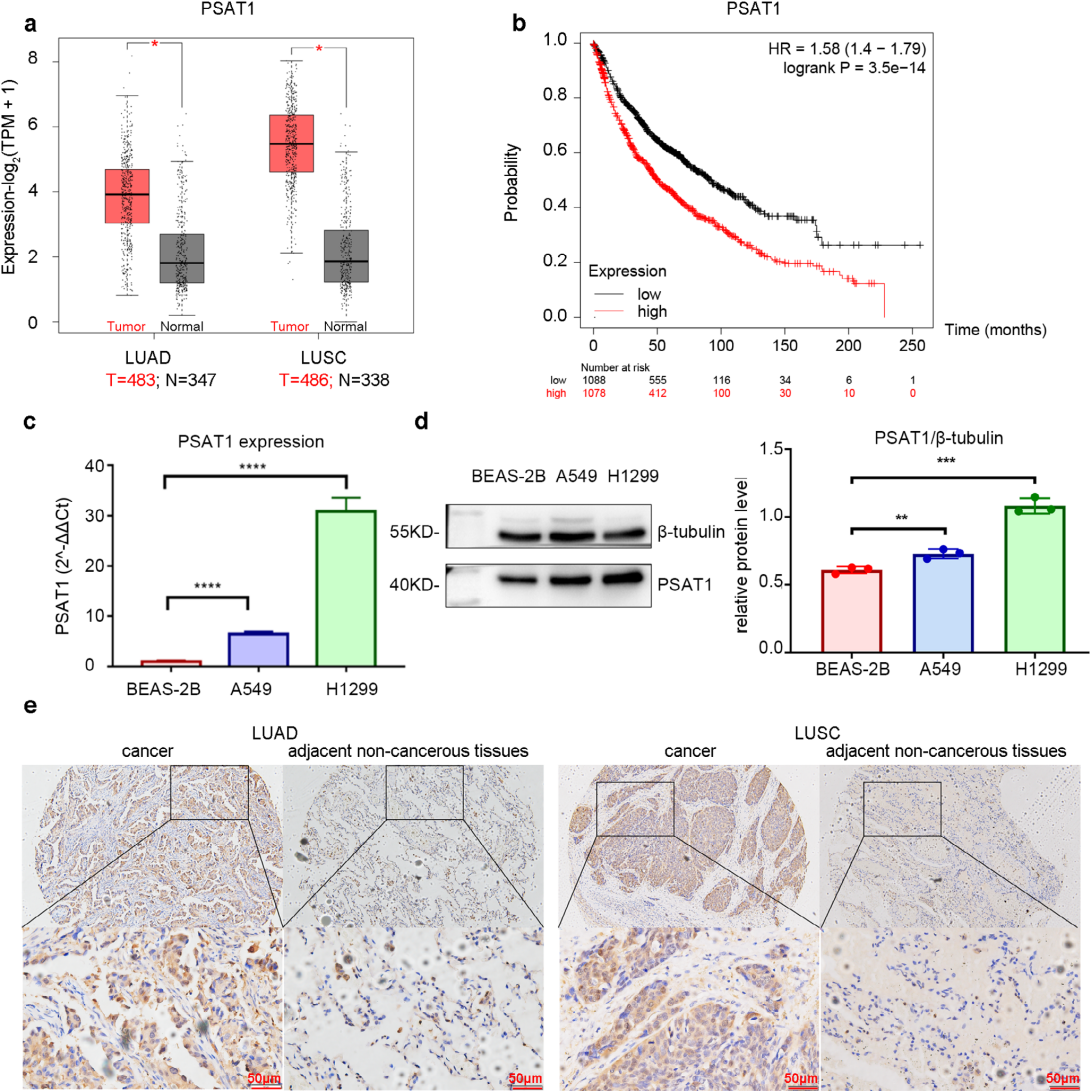
Expression of PSAT1 in NSCLC. (a) The expression of *PSAT1* is significantly upregulated in both LUAD and LUSC (LUAD: T = 483 *N* = 347, *p* < 0.05; LUSC: T = 486 *N* = 338, *p* < 0.05). (b) Patients with high *PSAT1* expression have a shorter OS time (log‐rank *p* < 0.05). (c, d) RT‐qPCR and WB experiments consistently demonstrate significantly elevated levels of *PSAT1* in NSCLC cell lines (H1299 and A549) compared to the bronchial epithelial cell line (BEAS‐2B) (*n* = 3, *p* < 0.05). (e) IHC results reveal a significant increase in *PSAT1* expression in LUAD and LUSC tissues compared to adjacent non‐cancerous tissues.

Bioinformatics analysis showed significantly elevated *PSAT1* expression in NSCLC vs. normal tissues (Figure 1a, LUAD: T = 483 *N* = 347, *p* < 0.05; LUSC: T = 486 *N* = 338, *p* < 0.05), correlating with poorer prognosis (Figure 1b, log‐rank *p* < 0.05). RT‐qPCR and WB confirmed higher *PSAT1* expression in H1299 and A549 cells compared to BEAS‐2B at both mRNA and protein levels (Figure 1c,d, *p* < 0.05).

IHC showed deeper staining in NSCLC compared to para‐cancerous tissues (Figure 1e), with a statistically significant increase observed in *PSAT1* expression within the former group (*p* < 0.05) (Table 1). Further analysis suggested an association between *PSAT1* expression and histological types of NSCLC (*p* < 0.05), while no significant correlations were found with age, sex, or TNM stage (Table 2).

**TABLE 1:** *PSAT1* expression levels in NSCLC and adjacent non‐cancerous tissues.

Characteristics	Numbers	*PSAT1*‐high	*PSAT1*‐low	*P* value
NSCLC	84	65	19	
Adjacent non‐cancerous tissues	86	18	68	< 0.05

**TABLE 2:** Association between *PSAT1* expression levels and clinicopathological characteristics of NSCLC patients.

Characteristics	Numbers	*PSAT1*‐high	*PSAT1*‐low	*P* value
Histological types				
LUSC	37	33	4	
LUAD	34	23	11	0.026
Age (years)				
≤ 60	38	28	10	
> 60	44	36	8	0.375
Gender				
Female	27	18	9	
Male	54	45	9	0.089
Tumor size				
< 5 cm	43	35	8	
≥ 5 cm	26	19	7	0.417
TNM stage				
I–II	8	6	2	
II–IV (include II)	55	42	13	0.933

*Abbreviations:* LUAD, lung adenocarcinoma; LUSC, lung squamous cell carcinoma; NSCLC, non–small cell lung cancer; TNM, tumor–node–metastasis.

### 3.2 Knockdown of PSAT1 Inhibits the Progression of NSCLC

**FIGURE 2:**
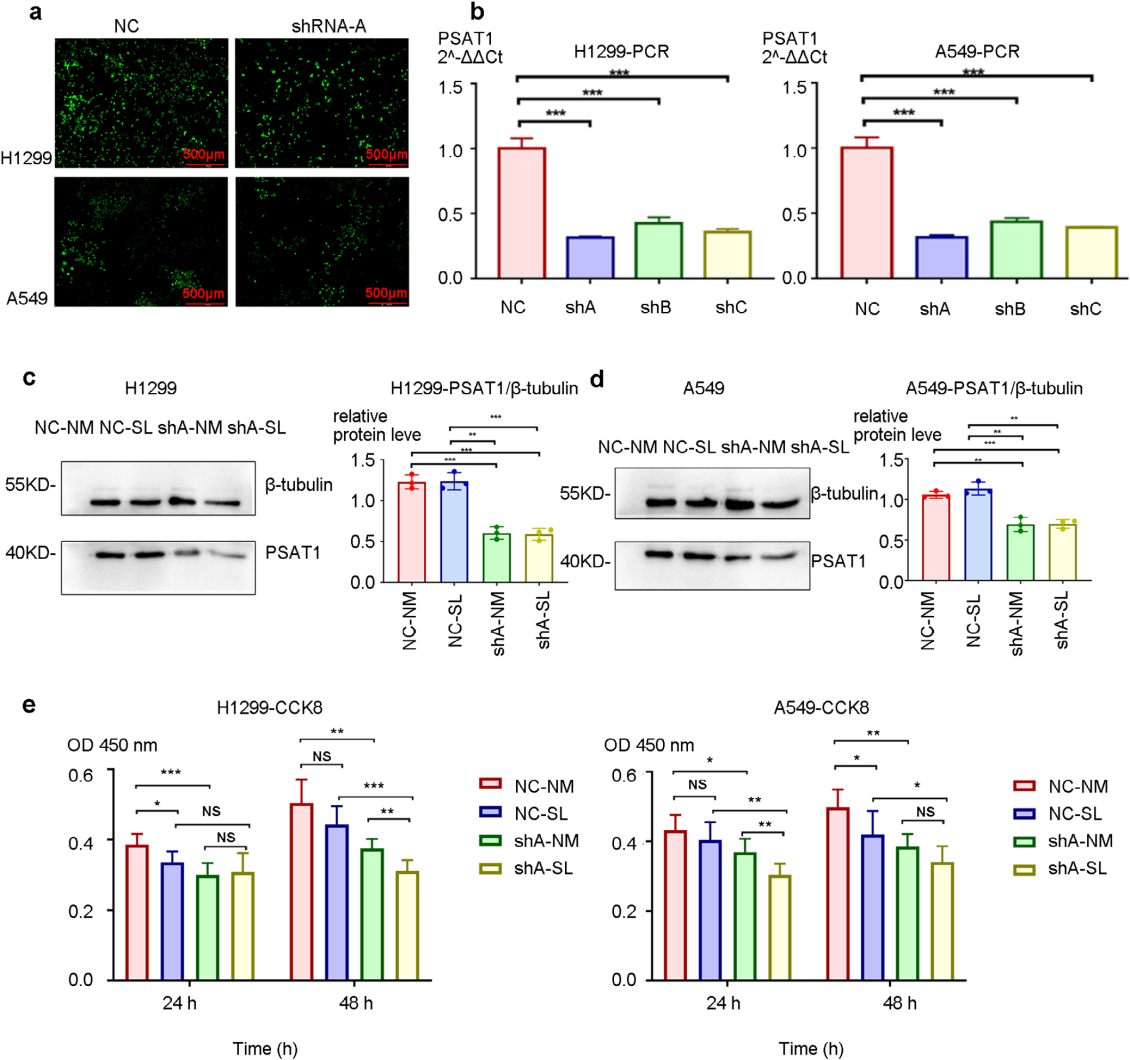
Construction of knockdown PSAT1 cell lines and the effect of knocking down PSAT1 on proliferation. Green fluorescence confirmed successful plasmid transfection. (b) shRNA‐A achieved superior *PSAT1* knockdown vs. shRNA‐B/C. (c, d) The lack of exogenous serine does not reduce the knockdown efficiency of lentivirus plasmids on *PSAT1* (*n* = 3, *p* < 0.05). (e) CCK‐8 experiments demonstrate that inhibition of NSCLC proliferation is achieved through *PSAT1* knockdown, which can be further enhanced by inhibiting exogenous serine intake (*n* = 6, *p* < 0.05).

We established stable *PSAT1*‐knockdown H1299 and A549 cells using optimal shRNA (Figure 2a,b). Serine‐deficient medium was used to assess *PSAT1* function under limited exogenous serine conditions. WB experiment confirmed serine restriction didn't affect shRNA knockdown efficiency (Figure 2c,d).

**FIGURE 3:**
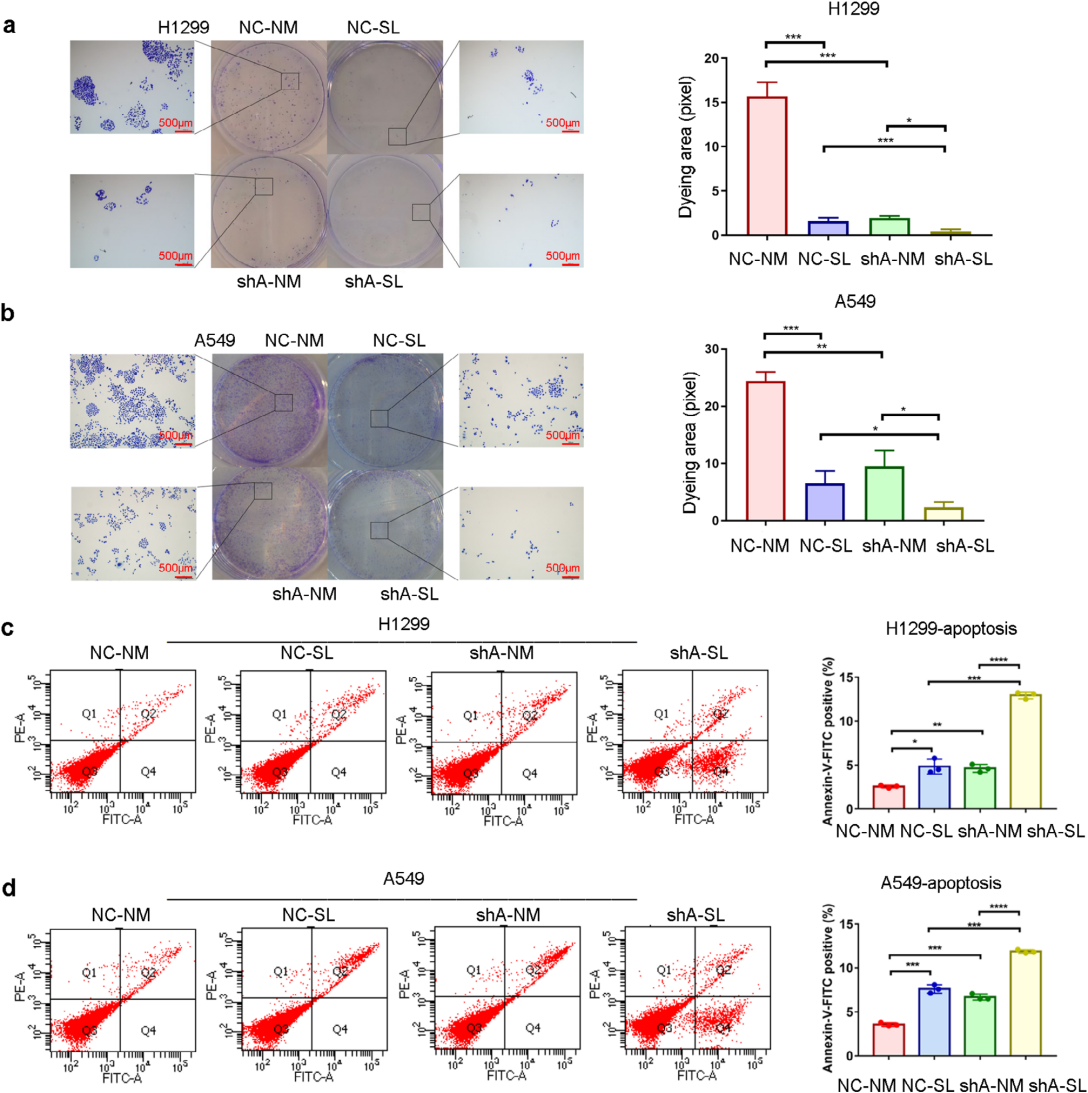
The impact of PSAT1 knockdown on cell proliferation and apoptosis. (a, b) Colony formation experiments demonstrate that knocking down *PSAT1* suppresses NSCLC proliferation, while reducing exogenous serine intake can enhance this inhibitory effect (*n* = 3, *p* < 0.05). (c, d) Flow cytometry results suggest that knocking down *PSAT1* promotes apoptosis in NSCLC cells. Insufficient exogenous serine intake enhances the apoptotic effect of *PSAT1* knockdown (*n* = 3, *p* < 0.05).

**FIGURE 4:**
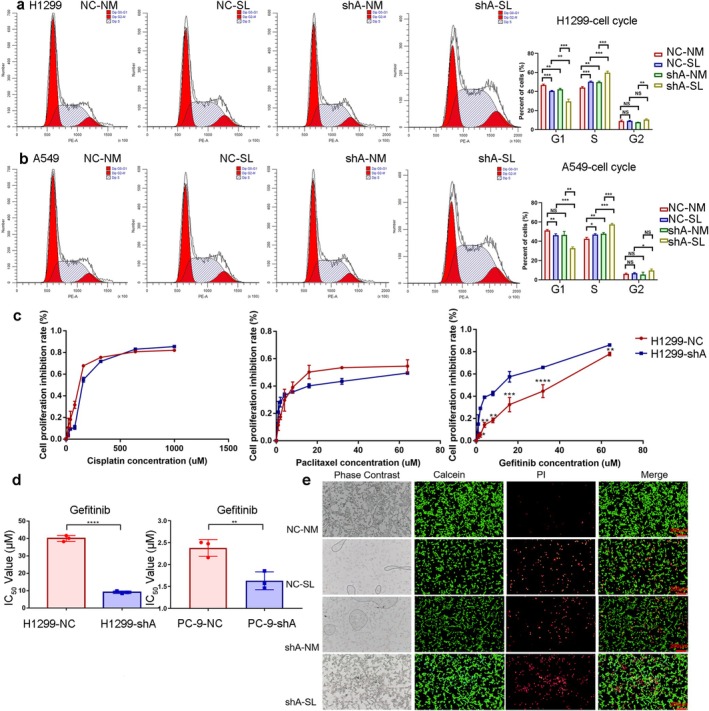
The influence of PSAT1 knockdown on cell cycle and therapeutic efficacy of gefitinib. (a, b) Inadequate exogenous serine intake and *PSAT1* knockdown regulate the cell cycle of NSCLC, resulting in an increase in S phase cells and a decrease in G1 phase cells (*n* = 3, *p* < 0.05). (c, d) Compared to the NC group, gefitinib exhibits stronger tumor‐killing effects in H1299 cells with stable *PSAT1* knockdown (*n* = 3, *p* < 0.05). (e) Calcein/PI staining results indicate that combining *PSAT1* knockdown with gefitinib leads to enhanced tumor‐killing effects. Moreover, reducing exogenous serine intake further enhances this effect.

*PSAT1* knockdown and serine deprivation independently reduced NSCLC cell proliferation (CCK‐8 and colony formation assays), with combined treatment showing maximal inhibition (Figure 2e, *n* = 6, *p* < 0.05; Figure 3a,b, *n* = 3, *p* < 0.05). Both interventions increased apoptosis, peaking in the dual‐treated group (shA‐SL) (Figure 3c,d). Cell cycle analysis revealed that *PSAT1* knockdown plus serine deprivation decreased G1 phase while increasing S phase populations (Figure 4a,b). To address potential off‐target effects, the key proliferation phenotype was validated using two additional independent shRNAs (shB and shC), which yielded consistent results (Figure S2). These results demonstrate that *PSAT1* ablation synergizes with serine restriction to suppress proliferation.

### 3.3 Knockdown of PSAT1 Enhances the Inhibitory Effect of Gefitinib on NSCLC

The CCK‐8 assay of the transfected H1299 cells treated with varying concentrations of gefitinib revealed that knockdown of *PSAT1* significantly enhanced the percentage of cell proliferation inhibition. However, this synergistic effect was not observed in the cisplatin and paclitaxel groups (Figure 4c, at 1–64 μM gefitinib, *n* = 3, *p < 0.05*). In H1299 cells with stable *PSAT1* knockdown, the half‐maximal inhibitory concentration (IC50) of gefitinib significantly decreased. To address the role of Epidermal Growth Factor Receptor (EGFR) mutation status, we also tested the EGFR‐mutant, gefitinib‐sensitive cell line PC‐9. *PSAT1* knockdown similarly reduced gefitinib IC50 in PC‐9 cells (Figure 4d, *p* < 0.05), demonstrating that the chemosensitizing effect extends to this clinically relevant genetic background (Figure 4d, *n* = 3, *p < 0.05*). Calcein/PI staining demonstrated that both serine deficiency and *PSAT1* knockdown promoted cell death when exposed to the same concentration of gefitinib. Among all groups, the shA‐SL group exhibited the highest rate of cellular mortality (Figure 4e).

### 3.4 Knockdown of PSAT1 Increases ROS Levels and Upregulates the Mitochondrial Apoptotic Pathway

**FIGURE 5:**
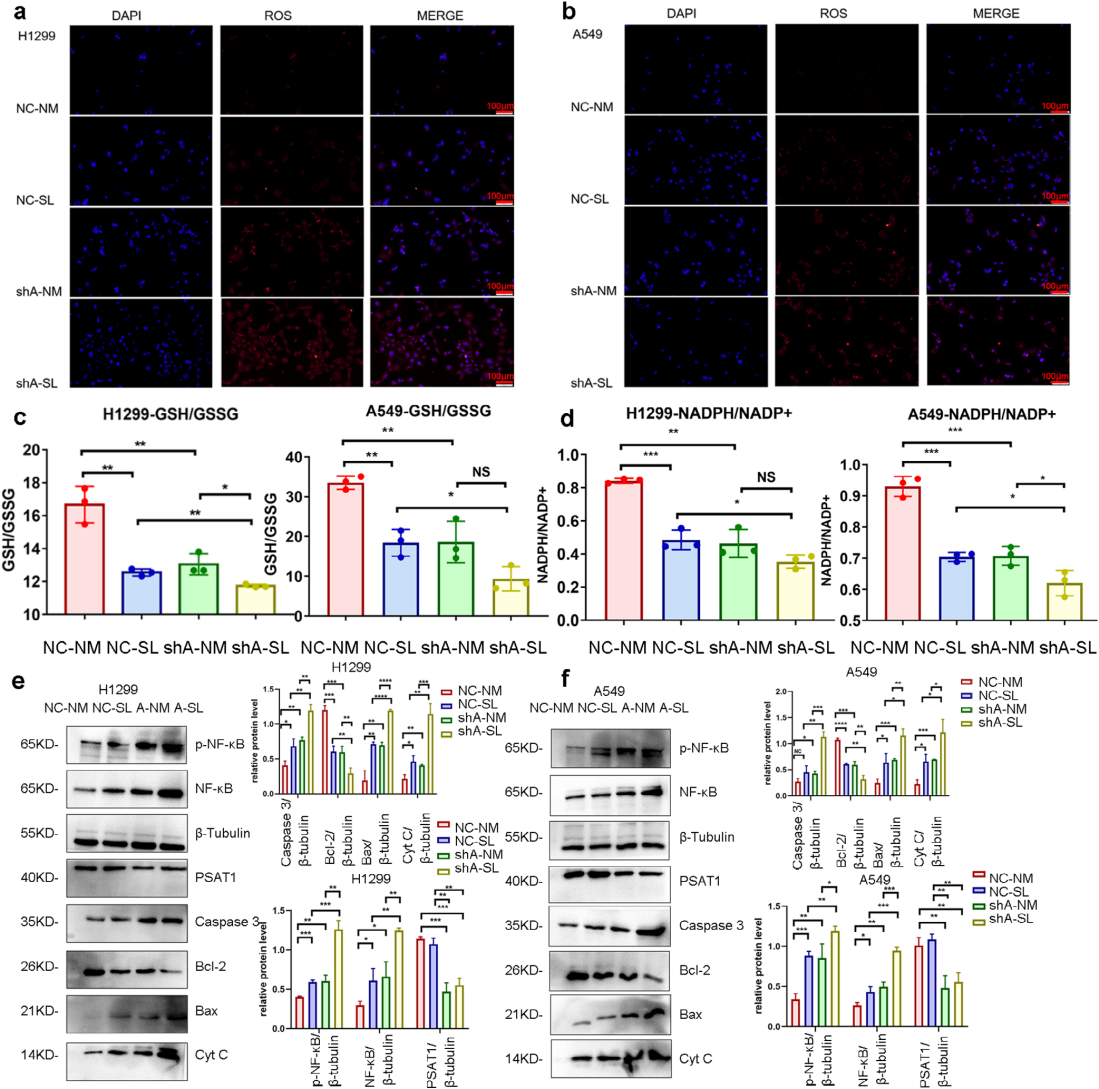
The impact of PSAT1 knockdown on cellular redox balance. (a, b) Both *PSAT1* knockdown and serine restriction increased ROS levels in NSCLC cells. (c, d) Detection of GSH/GSSG ratio and NADPH/NADP+ ratio confirms the elevation of intracellular ROS caused by *PSAT1* knockdown and insufficient serine intake (*n* = 3, *p* < 0.05). (e, f) WB analysis showed serine deficiency activated NF‐κB and mitochondrial apoptosis pathways, with enhanced effects under combined endogenous/exogenous serine deprivation (*n* = 3, *p* < 0.05).

ROS levels were significantly elevated in both NC‐SL and shA‐NM groups vs. NC‐NM controls, with shA‐SL showing maximal fluorescence intensity (Figure 5a,b, *n* = 3, *p < 0.05*). Additionally, the augmentation of intracellular levels of ROS in the experiment can be attenuated by ROS scavengers, such as N‐Acetyl‐L‐cysteine (NAC) (Figure S3, S4). This result was consistent with the GSH/GSSG and NADPH/NADP+ ratio detection (Figure 5c,d, *n* = 3, *p < 0.05*), indicating that *PSAT1* knockdown and inadequate serine intake can lead to an elevation in intracellular ROS.

The WB results demonstrated a significant increase in p‐NF‐κB and NF‐κB expression in both the NC‐SL and shA‐NM groups compared to the NC‐NM group. Additionally, activation of the mitochondrial pathway of apoptosis was observed in these two groups, characterized by decreased Bcl‐2 expression and increased expression of Bax, Cyt C, and Caspase 3. Notably, among all four groups, the shA‐SL group displayed the highest expression of p‐NF‐κB and NF‐κB, along with pronounced activation of the mitochondrial apoptotic pathway (Figure 5e,f, *n* = 3, *p < 0.05*). To directly confirm the execution of apoptosis, we quantified cells containing active caspase‐3 using flow cytometry. Consistent with the WB trends, both serine deprivation (NC‐SL) and *PSAT1* knockdown (shA‐NM) significantly increased the population of active caspase‐3‐positive cells, with the combined treatment (shA‐SL) showing the highest level (Figure S6, *n* = 3, *p* < 0.05).

To further establish the causal role of NF‐κB pathway activation in this process, we conducted rescue experiments. Treatment of *PSAT1*‐knockdown cells under serine deprivation (shA‐SL) with the NF‐κB inhibitor BAY 11–7082 significantly reduced the percentage of apoptotic cells. Concurrently, the elevated ROS levels observed in shA‐SL cells were also partially attenuated upon NF‐κB inhibition (Figure S5, *n* = 3, *p < 0.05*). These rescue experiments demonstrate that NF‐κB activation is not merely correlative but functionally essential for driving the apoptotic response triggered by *PSAT1* ablation and serine deficiency.

### 3.5 Knockdown of PSAT1 Inhibits SSP in NSCLC

**FIGURE 6:**
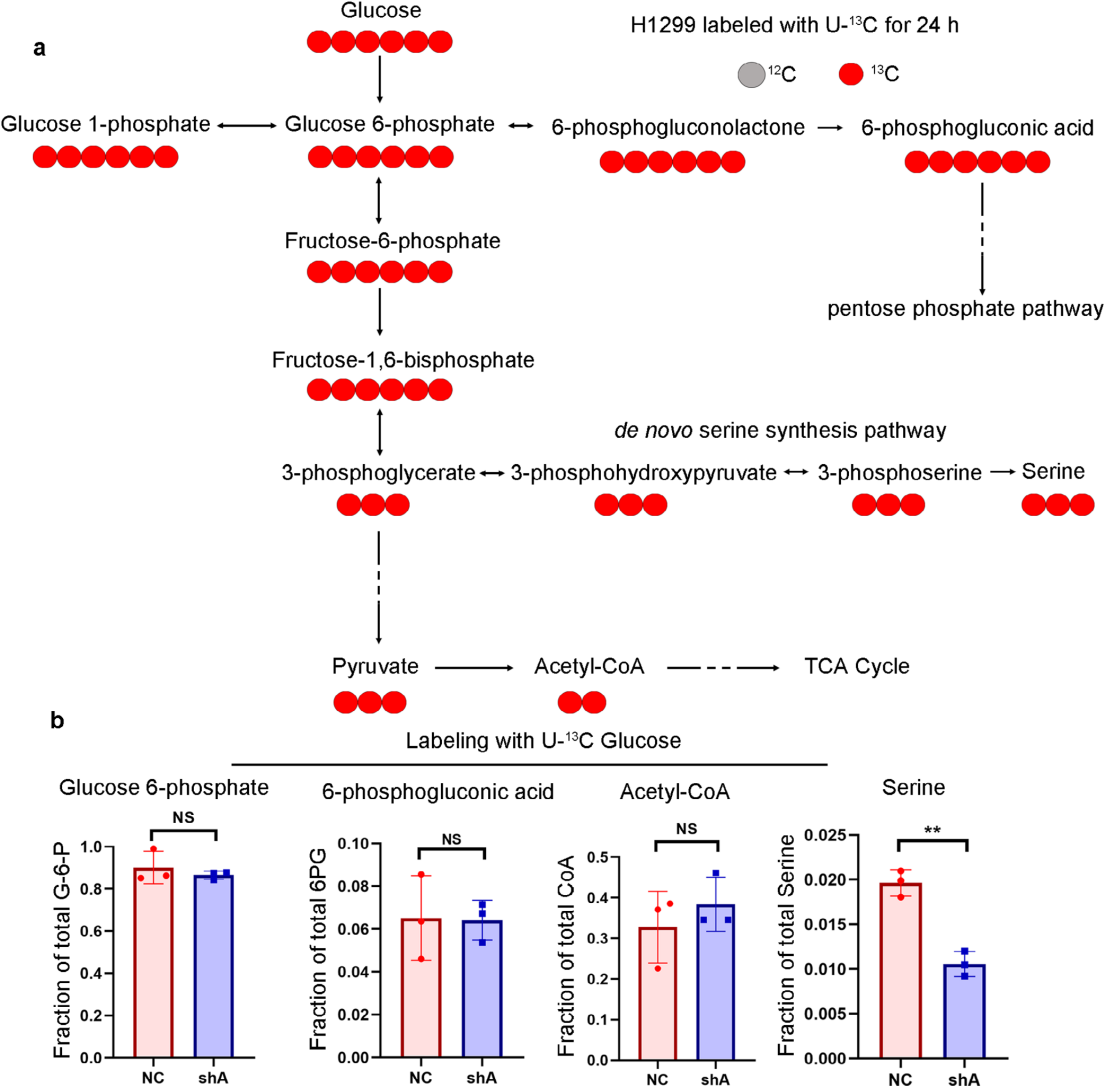
The mechanism of PSAT1 identified by U‐^13^C‐labeled glucose. (a) Utilizing U‐^13^C‐labeled glucose to elucidate glucose metabolism flux within the H1299 cell line. (b) By measuring the fraction of metabolites, it can be inferred that knocking down *PSAT1* primarily affects glucose entry into SSP, without significantly impacting the TCA cycle and the pentose phosphate pathway (*n* = 3, *p* < 0.05).

To assess the impact of *PSAT1* inhibition on endogenous serine metabolism, H1299 cells and A549 cells were cultured in RPMI 1640 medium supplemented with U‐^13^C‐labeled glucose instead of unlabeled glucose, and the fraction of metabolites was quantified. Additionally, we investigated other glucose metabolic pathways to elucidate the influence of *PSAT1* on glucose metabolism (Figure 6a and Figures S7, S8). A substantial quantity of M + 6 glucose 6‐phosphate was detected in both the NC and shA groups, indicating successful metabolism of U‐^13^C‐labeled glucose in NSCLC cells. The proportion of M + 3 serine derived from U‐^13^C‐labeled glucose was significantly lower in the shA group compared to the NC group, providing further evidence that *PSAT1* knockdown effectively inhibits SSP. Moreover, there were no significant differences observed in the fractions of 6‐phosphogluconic acid and acetyl‐CoA between the NC and shA groups, suggesting that knocking down *PSAT1* does not exert a notable impact on the TCA cycle and the pentose phosphate pathway (Figure 6b and Figure S8).

### 3.6 Knockdown of PSAT1 Inhibits the Growth of NSCLC in Nude Mice

**FIGURE 7:**
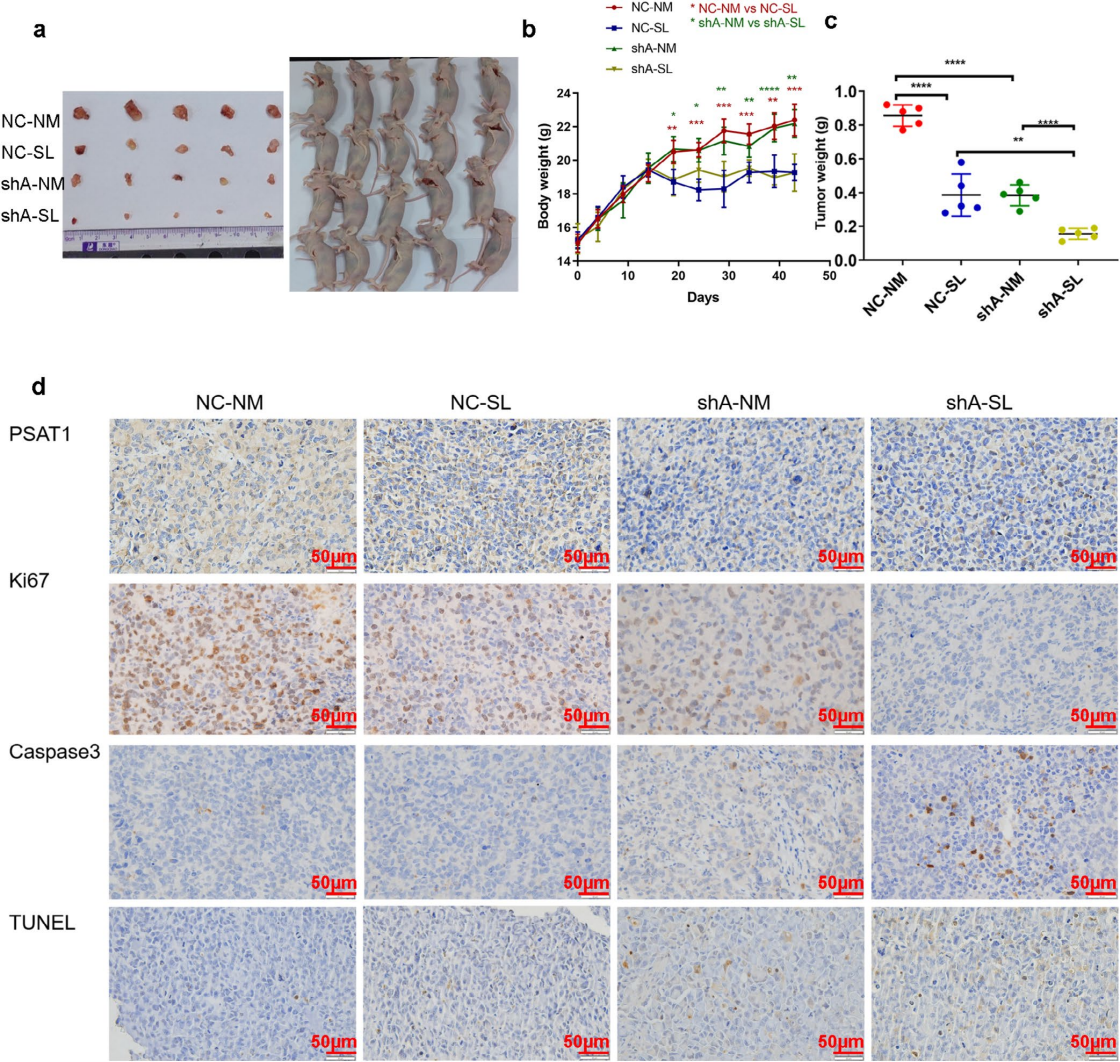
The function of PSAT1 in xenograft models. (a) Combined endogenous (*PSAT1* knockdown) and exogenous (serine‐deficient diet) serine reduction synergistically inhibited NSCLC tumor growth. (b) Mice receiving serine‐deficient diets (NC‐SL, shA‐SL) showed significant weight loss (*n* = 5, *p* < 0.05). (c) Tumor burden was lowest in the shA‐SL group (*n* = 5, *p* < 0.05). (d) IHC confirmed *PSAT1* knockdown (shA groups), with Ki67 staining showing reduced proliferation and Caspase 3/TUNEL staining indicating increased apoptosis under serine deprivation.

Tumor weights and volumes were significantly reduced in NC‐SL and shA‐NM groups vs. NC‐NM controls, with shA‐SL showing the most pronounced inhibition (Figure 7a–c, *n* = 5, *p < 0.05*). From day 14, the serine‐deficient diet (NC‐SL and shA‐SL) attenuated mouse weight gain. IHC confirmed *PSAT1* downregulation in shA groups, with Ki67 revealing maximal proliferation in the NC‐NM group and minimal in the shA‐SL group. Caspase 3 and TUNEL staining demonstrated apoptotic activity specifically in shA‐SL tumors (Figure 7d).

## 4 Discussion

Here, we propose that *PSAT1* plays a key regulatory role in serine metabolism in NSCLC. In addition to regulating the proliferation and apoptosis of NSCLC cells, *PSAT1* knockdown enhances the killing effect of gefitinib on NSCLC cells. *PSAT1*, as the second key enzyme in SSP, catalyzes the conversion of 3‐phosphohydroxypyruvate (3‐PP) to 3‐phosphoserine (3‐PS). Although *PSAT1* is not the first key enzyme in the SSP pathway, no known metabolic pathway bypasses *PSAT1* to achieve serine synthesis downstream of *PHGDH* [16]. Inhibition of *PSAT1* can reduce the synthesis of endogenous serine, similar to the inhibition of *PHGDH* [22]. Our isotope‐tracing experimental results also demonstrate that knocking down *PSAT1* inhibited the synthesis of serine through SSP in NSCLC. According to our survey of published literature, many studies on targeted drugs for *PHGDH* are available, while research on *PSAT1* needs to progress. Moreover, our study demonstrates that *PSAT1* is significantly overexpressed in NSCLC. Therefore, targeting *PSAT1* may provide a direction for the development of targeted drugs for NSCLC.

The mechanism by which *PSAT1* influences NSCLC cell proliferation and apoptosis involves its essential role in maintaining serine homeostasis. Serine is not only a raw material for protein synthesis, but also an essential source of 1C units [26]. Serine hydroxymethyl transferase (SHMT) catalyzes the production of glycine from serine and simultaneously transfers the γ‐carbon amino acid side chain to tetrahydrofolic acid (THF) to generate 5, 10‐methylene‐THF (me‐THF). Me‐THF is further converted into formyl‐THF, sustaining purine nucleotide synthesis [27, 28]. We propose that in the absence of serine, nucleotide synthesis in NSCLC cells is inhibited, the cell cycle is blocked, and cell proliferation is weakened. It is worth noting that, although serine and glycine can be converted into each other in the human body, the process of converting glycine to serine consumes 1C units. Excessive glycine may occasionally be harmful to cancer cell proliferation [29]. Therefore, even if serine and glycine can be interconverted, SSP and exogenous uptake of serine are still necessary for the rapid proliferation of tumor cells.

Serine stimulates GSH synthesis, which is crucial for maintaining intracellular redox levels [30]. When endogenous and exogenous serine levels are reduced, GSH synthesis is insufficient and intracellular ROS levels increase [31]. ROS are produced via various cellular processes and serve as important molecules in cellular signaling events [32]. Previous studies have shown that moderate amounts of ROS promote tumor cell growth, while significantly increased ROS levels induce cell apoptosis [33, 34]. The regulatory role of ROS in various biological reaction processes is complex and extensive. For example, studies have suggested a crosstalk between ROS and the NF‐κB signaling pathway [32]. An increase in ROS levels may lead to the upregulation of protein levels related to the NF‐κB signaling pathway. An increase in ROS levels can also induce crosstalk between AMPK and the AKT pathway [35]. Excessive ROS production causes mitochondrial damage and nuclear DNA mutations, which lead to further ROS production and a vicious cycle of mitochondrial, ROS, and genomic instability [36]. In the absence of serine, flow cytometry detected increased cell apoptosis, and WB indicated activation of the mitochondrial apoptotic pathway. Therefore, we propose that *PSAT1* knockdown perturbs glutathione synthesis, leading to intracellular ROS accumulation. This oxidative stress, in turn, activates the NF‐κB pathway, which is a critical contributor to the ensuing apoptosis. This inference is supported by our observation that inhibition of NF‐κB with BAY 11–7082 significantly attenuated both ROS elevation and apoptosis in *PSAT1*‐deficient cells under serine deprivation. Collectively, these findings indicate that the ROS–NF‐κB axis plays an essential role in executing mitochondrial apoptotic pathways upon serine metabolic disruption.

Previous studies indicate that serine deprivation activates *ATF4*/*ATF3*, upregulating SSP enzymes like *PHGDH* and *PSAT1* [13, 37]. We found that when the intake of exogenous serine was reduced, the inhibitory effect of lentiviral plasmids on *PSAT1* expression could be maintained. Moreover, simultaneous *PSAT1* knockdown and exogenous serine deficiency achieved superior inhibitory effects on the progression of NSCLC. While current NSCLC treatments include surgery, radiation, chemotherapy, and targeted therapy [38], metabolic targeting has emerged as a promising strategy [39]. Notably, *PSAT1* inhibition reverses erlotinib resistance in lung adenocarcinoma [40]. Similar to erlotinib, gefitinib is an oral *EGFR* inhibitor used clinically to treat patients with NSCLC with *EGFR*‐sensitive mutations (exon 19 deletion or L858R point mutation) [41]. In phase II clinical trials, oral gefitinib improved symptoms in patients with NSCLC who had previously received chemotherapy [42]. The therapeutic effect of gefitinib is more obvious in never‐smokers, women, and Asian patients, with higher proportions of specific *EGFR* mutations [43]. Our experimental results indicated that *PSAT1* knockdown enhanced gefitinib's antitumor efficacy but showed no significant effect on paclitaxel or cisplatin. We propose that inhibiting SSP and reducing serine uptake can be used as part of the metabolic therapy for NSCLC and may lead to better efficacy in combination therapy with targeted drugs such as gefitinib.

It is important to note that our functional studies primarily utilized H1299 (EGFR wild‐type, p53 null) and A549 (*EGFR* wild‐type, *KRAS* mutant) cell lines. These specific genetic backgrounds—particularly the absence of canonical *EGFR* sensitizing mutations—likely influenced the magnitude of gefitinib sensitization observed. Our subsequent validation using the *EGFR*‐mutant PC‐9 cell line confirmed that *PSAT1* knockdown also enhances gefitinib sensitivity in this genetic context. Therefore, while our data suggest that the effect of *PSAT1* inhibition may extend across different *EGFR* statuses, caution is still warranted when extrapolating the underlying mechanisms to all NSCLC subtypes, given the heterogeneity in concurrent genetic alterations. Future validation in a broader panel of cell lines remains valuable.

Enhancement of serine synthesis is an adaptive mechanism for the malignant proliferation of tumor cells, which represents a metabolic vulnerability in NSCLC. Inhibiting *PSAT1* to attenuate endogenous serine synthesis can enhance the therapeutic efficacy of targeted drugs for NSCLC. A limitation of our study is the use of adjacent non‐cancerous tissues as IHC controls, which may not fully represent the normal bronchial epithelium. Additionally, our work focused on tumor growth and drug sensitivity; the role of *PSAT1* in NSCLC invasion and metastasis remains unexplored and represents a key direction for future research. The dietary serine restriction in our mouse model, while informative, may not fully recapitulate the metabolic stress in human tumors. Lastly, the clinical sample size, though statistically informative, could be expanded in future studies to strengthen correlative findings. Further investigation is warranted to elucidate the mechanism underlying aberrant serine metabolism and facilitate drug development. Furthermore, our retrospective clinical cohort lacked comprehensive molecular profiling (e.g., *EGFR*/*ALK* mutation status) and detailed treatment histories, which precluded robust correlative analyses between *PSAT1* expression and specific drug resistance patterns or patient subgroups. While the functional synergy between *PSAT1* knockdown and gefitinib was mechanistically explored in vitro, validating this association in larger, prospectively annotated clinical cohorts is essential. Finally, although this study elucidates the *PSAT1*/SSP‐ROS‐NF‐κB apoptotic axis, translating this vulnerability into therapeutic strategies—such as developing specific PSAT1 inhibitors or optimizing dietary serine restriction regimens—requires substantial future investigation.

## Author Contributions

**Yingshu Cao:** data curation, writing – review and editing. **Xiangyu Zhou:** data curation, writing – review and editing. **Xijia Zhou:** conceptualization, writing – original draft, data curation, formal analysis, investigation, methodology, visualization. **Min Zhao:** data curation, formal analysis, writing – review and editing. **Ke Wang:** conceptualization, funding acquisition, methodology, writing – review and editing.

## Funding

This work was supported by the “Medical+X” Cross Innovation Team, “Unveiling and Leading” Construction Project, 2022JBGS07. Disciplinary Crossing and Integration and Innovation Cultivation Project of Jilin University, JLUXKJC2020212.

## Ethics Statement

This study was approved by the Ethics Committee of the Second Hospital of Jilin University and the Institutional Animal Care and Use Committee of Jilin University. Informed consent was obtained from all subjects and/or their legal guardian(s). All methods are reported in accordance with ARRIVE guidelines (https://arriveguidelines.org) for the reporting of animal experiments.

## Conflicts of Interest

The authors declare no conflicts of interest.

## Supporting information


**Data S1:** Supporting Information.

**Figure S1:** Scoring standards of IHC staining.
**Figure S2:** CCK‐8 assay conducted on the H1299 cell line transfected with shB and shC.
**Figure S3:** Original figures of ROS.
**Figure S4:** Original figures of ROS (adding ROS scavengers NAC).
**Figure S5:** Inhibition of NF‐κB rescues apoptosis and attenuates ROS elevation induced by PSAT1 knockdown under serine deprivation.
**Figure S6:** Flow cytometric analysis of cleaved caspase‐3 in H1299 cells.
**Figure S7:** All maps of the isotope labeling experiment.
**Figure S8:** Isotope labeling experiment using the A549 cell line.

## Data Availability

The data that support the findings of this study are available from the corresponding author upon reasonable request.

## References

[1] R. L. Siegel , A. N. Giaquinto , and A. Jemal , “Cancer Statistics,” CA: A Cancer Journal for Clinicians 74, no. 1 (2024): 12–49. 38230766 10.3322/caac.21820

[2] K. D. Miller , L. Nogueira , T. Devasia , et al., “Cancer Treatment and Survivorship Statistics,” CA: A Cancer Journal for Clinicians 72, no. 5 (2022): 409–436. 35736631 10.3322/caac.21731

[3] K. Bajbouj , A. Al‐Ali , R. K. Ramakrishnan , M. Saber‐Ayad , and Q. Hamid , “Histone Modification in NSCLC: Molecular Mechanisms and Therapeutic Targets,” International Journal of Molecular Sciences 22, no. 21 (2021): 11701. 34769131 10.3390/ijms222111701 PMC8584007

[4] D. S. Ettinger , D. E. Wood , D. L. Aisner , et al., “Non‐Small Cell Lung Cancer, Version 3.2022, NCCN Clinical Practice Guidelines in Oncology,” Journal of the National Comprehensive Cancer Network 20, no. 5 (2022): 497–530. 35545176 10.6004/jnccn.2022.0025

[5] M. Wang , R. S. Herbst , and C. Boshoff , “Toward Personalized Treatment Approaches for Non‐Small‐Cell Lung Cancer,” Nature Medicine 27, no. 8 (2021): 1345–1356. 10.1038/s41591-021-01450-2 34385702

[6] B. Faubert , A. Solmonson , and R. J. DeBerardinis , “Metabolic Reprogramming and Cancer Progression,” Science 368, no. 6487 (2020): eaaw5473. 32273439 10.1126/science.aaw5473 PMC7227780

[7] K. Yang , X. Wang , C. Song , et al., “The Role of Lipid Metabolic Reprogramming in Tumor Microenvironment,” Theranostics 13, no. 6 (2023): 1774–1808. 37064872 10.7150/thno.82920 PMC10091885

[8] M. G. Vander Heiden , L. C. Cantley , and C. B. Thompson , “Understanding the Warburg Effect: The Metabolic Requirements of Cell Proliferation,” Science 324, no. 5930 (2009): 1029–1033. 19460998 10.1126/science.1160809 PMC2849637

[9] P. Vaupel and G. Multhoff , “Revisiting the Warburg Effect: Historical Dogma Versus Current Understanding,” Journal of Physiology 599, no. 6 (2021): 1745–1757. 33347611 10.1113/JP278810

[10] L. N. Raines , H. Zhao , Y. Wang , et al., “PERK Is a Critical Metabolic Hub for Immunosuppressive Function in Macrophages,” Nature Immunology 23, no. 3 (2022): 431–445. 35228694 10.1038/s41590-022-01145-x PMC9112288

[11] L. He , J. Endress , S. Cho , et al., “Suppression of Nuclear GSK3 Signaling Promotes Serine/One‐Carbon Metabolism and Confers Metabolic Vulnerability in Lung Cancer Cells,” Science Advances 8, no. 20 (2022): eabm8786. 35594343 10.1126/sciadv.abm8786 PMC9122323

[12] W. Yu , Z. Wang , K. Zhang , et al., “One‐Carbon Metabolism Supports S‐Adenosylmethionine and Histone Methylation to Drive Inflammatory Macrophages,” Molecular Cell 75, no. 6 (2019): 1147–1160.e1145. 31420217 10.1016/j.molcel.2019.06.039

[13] D. Zhang , A. M. Li , G. Hu , et al., “PHGDH‐Mediated Endothelial Metabolism Drives Glioblastoma Resistance to Chimeric Antigen Receptor T Cell Immunotherapy,” Cell Metabolism 35, no. 3 (2023): 517–534.e518. 36804058 10.1016/j.cmet.2023.01.010 PMC10088869

[14] S. Krieg , S. I. Fernandes , C. Kolliopoulos , M. Liu , and S. M. Fendt , “Metabolic Signaling in Cancer Metastasis,” Cancer Discovery 14, no. 6 (2024): 934–952. 38592405 10.1158/2159-8290.CD-24-0174 PMC7616057

[15] A. E. Rodriguez , G. S. Ducker , L. K. Billingham , et al., “Serine Metabolism Supports Macrophage IL‐1beta Production,” Cell Metabolism 29, no. 4 (2019): 1003–1011e1004. 30773464 10.1016/j.cmet.2019.01.014 PMC6447453

[16] A. Buqué , L. Galluzzi , and D. C. Montrose , “Targeting Serine in Cancer: Is Two Better Than One?,” Trends Cancer 7, no. 8 (2021): 668–670. 34219053 10.1016/j.trecan.2021.06.004 PMC9097339

[17] K. Wang , L. Luo , S. Fu , et al., “PHGDH Arginine Methylation by PRMT1 Promotes Serine Synthesis and Represents a Therapeutic Vulnerability in Hepatocellular Carcinoma,” Nature Communications 14, no. 1 (2023): 1011. 10.1038/s41467-023-36708-5 PMC9950448 36823188

[18] T. Muthusamy , T. Cordes , M. K. Handzlik , et al., “Serine Restriction Alters Sphingolipid Diversity to Constrain Tumour Growth,” Nature 586, no. 7831 (2020): 790–795. 32788725 10.1038/s41586-020-2609-x PMC7606299

[19] X. Wang , S. Min , H. Liu , et al., “Nf1 Loss Promotes Kras‐Driven Lung Adenocarcinoma and Results in Psat1‐Mediated Glutamate Dependence,” EMBO Molecular Medicine 11, no. 6 (2019): e9856. 31036704 10.15252/emmm.201809856 PMC6554671

[20] P. A. Stewart , E. A. Welsh , R. J. C. Slebos , et al., “Proteogenomic Landscape of Squamous Cell Lung Cancer,” Nature Communications 10, no. 1 (2019): 3578. 10.1038/s41467-019-11452-x PMC6687710 31395880

[21] Y. Yang , J. Wu , J. Cai , et al., “PSAT1 Regulates Cyclin D1 Degradation and Sustains Proliferation of Non‐Small Cell Lung Cancer Cells,” International Journal of Cancer 136, no. 4 (2015): E39–E50. 25142862 10.1002/ijc.29150

[22] D. C. Montrose , S. Saha , M. Foronda , et al., “Exogenous and Endogenous Sources of Serine Contribute to Colon Cancer Metabolism, Growth, and Resistance to 5‐Fluorouracil,” Cancer Research 81, no. 9 (2021): 2275–2288. 33526512 10.1158/0008-5472.CAN-20-1541 PMC8137552

[23] M. J. Zheng , X. Li , Y. X. Hu , et al., “Identification of Molecular Marker Associated With Ovarian Cancer Prognosis Using Bioinformatics Analysis and Experiments,” Journal of Cellular Physiology 234, no. 7 (2019): 11023–11036. 30633343 10.1002/jcp.27926

[24] C. Zhang , J. J. Yu , C. Yang , et al., “Wild‐Type IDH1 Maintains NSCLC Stemness and Chemoresistance Through Activation of the Serine Biosynthetic Pathway,” Science Translational Medicine 15, no. 726 (2023): eade4113. 38091408 10.1126/scitranslmed.ade4113

[25] J. Zhang , X. Wang , V. Vikash , et al., “ROS and ROS‐Mediated Cellular Signaling,” Oxidative Medicine and Cellular Longevity 2016 (2016): 4350965. 26998193 10.1155/2016/4350965 PMC4779832

[26] S. L. Geeraerts , E. Heylen , K. De Keersmaecker , and K. R. Kampen , “The Ins and Outs of Serine and Glycine Metabolism in Cancer,” Nature Metabolism 3, no. 2 (2021): 131–141. 10.1038/s42255-020-00329-9 33510397

[27] A. M. Li and J. Ye , “Reprogramming of Serine, Glycine and One‐Carbon Metabolism in Cancer,” Biochimica et Biophysica Acta ‐ Molecular Basis of Disease 1866, no. 10 (2020): 165841. 32439610 10.1016/j.bbadis.2020.165841 PMC7442608

[28] J. C. García‐Cañaveras , O. Lancho , G. S. Ducker , et al., “SHMT Inhibition Is Effective and Synergizes With Methotrexate in T‐Cell Acute Lymphoblastic Leukemia,” Leukemia 35, no. 2 (2021): 377–388. 32382081 10.1038/s41375-020-0845-6 PMC7647950

[29] C. F. Labuschagne , N. J. van den Broek , G. M. Mackay , K. H. Vousden , and O. D. Maddocks , “Serine, but Not Glycine, Supports One‐Carbon Metabolism and Proliferation of Cancer Cells,” Cell Reports 7, no. 4 (2014): 1248–1258. 24813884 10.1016/j.celrep.2014.04.045

[30] H. Kurniawan , D. G. Franchina , L. Guerra , et al., “Glutathione Restricts Serine Metabolism to Preserve Regulatory T Cell Function,” Cell Metabolism 31, no. 5 (2020): 920–936.e927. 32213345 10.1016/j.cmet.2020.03.004 PMC7265172

[31] J. Muri and M. Kopf , “Redox Regulation of Immunometabolism,” Nature Reviews Immunology 21, no. 6 (2021): 363–381. 10.1038/s41577-020-00478-8 33340021

[32] M. J. Morgan and Z. G. Liu , “Crosstalk of Reactive Oxygen Species and NF‐κB Signaling,” Cell Research 21, no. 1 (2011): 103–115. 21187859 10.1038/cr.2010.178 PMC3193400

[33] J. D. Hayes , A. T. Dinkova‐Kostova , and K. D. Tew , “Oxidative Stress in Cancer,” Cancer Cell 38, no. 2 (2020): 167–197. 32649885 10.1016/j.ccell.2020.06.001 PMC7439808

[34] J. N. Moloney and T. G. Cotter , “ROS Signalling in the Biology of Cancer,” Seminars in Cell & Developmental Biology 80 (2018): 50–64. 28587975 10.1016/j.semcdb.2017.05.023

[35] Y. Zhao , X. Hu , Y. Liu , et al., “ROS Signaling Under Metabolic Stress: Cross‐Talk Between AMPK and AKT Pathway,” Molecular Cancer 16, no. 1 (2017): 79. 28407774 10.1186/s12943-017-0648-1 PMC5390360

[36] Y. Yang , S. Karakhanova , W. Hartwig , et al., “Mitochondria and Mitochondrial ROS in Cancer: Novel Targets for Anticancer Therapy,” Journal of Cellular Physiology 231, no. 12 (2016): 2570–2581. 26895995 10.1002/jcp.25349

[37] X. Li , D. Gracilla , L. Cai , et al., “ATF3 Promotes the Serine Synthesis Pathway and Tumor Growth Under Dietary Serine Restriction,” Cell Reports 36, no. 12 (2021): 109706. 34551291 10.1016/j.celrep.2021.109706 PMC8491098

[38] N. Duma , R. Santana‐Davila , and J. R. Molina , “Non–Small Cell Lung Cancer: Epidemiology, Screening, Diagnosis, and Treatment,” Mayo Clinic Proceedings 94, no. 8 (2019): 1623–1640. 31378236 10.1016/j.mayocp.2019.01.013

[39] Z. E. Stine , Z. T. Schug , J. M. Salvino , and C. V. Dang , “Targeting Cancer Metabolism in the Era of Precision Oncology,” Nature Reviews Drug Discovery 21, no. 2 (2022): 141–162. 34862480 10.1038/s41573-021-00339-6 PMC8641543

[40] M. Y. Luo , Y. Zhou , W. M. Gu , et al., “Metabolic and Nonmetabolic Functions of PSAT1 Coordinate Signaling Cascades to Confer EGFR Inhibitor Resistance and Drive Progression in Lung Adenocarcinoma,” Cancer Research 82, no. 19 (2022): 3516–3531. 36193649 10.1158/0008-5472.CAN-21-4074

[41] J. Rawluk and C. F. Waller , “Gefitinib,” Recent Results in Cancer Research 211 (2018): 235–246. 30069771 10.1007/978-3-319-91442-8_16

[42] T. Hida , S. Ogawa , J. C. Park , et al., “Gefitinib for the Treatment of Non‐Small‐Cell Lung Cancer,” Expert Review of Anticancer Therapy 9, no. 1 (2009): 17–35. 19105704 10.1586/14737140.9.1.17

[43] S. Dhillon , “Gefitinib: A Review of Its Use in Adults With Advanced Non‐Small Cell Lung Cancer,” Targeted Oncology 10, no. 1 (2015): 153–170. 25637458 10.1007/s11523-015-0358-9

