# Supplementary material for: PSAT1 Promotes NSCLC Progression via the De Novo Serine Synthesis Pathway and Represents a Therapeutic Vulnerability
Source: Cancer Med. 2026 Apr 2;15(4):e71780. doi: 10.1002/cam4.71780 (PMC13045451; doi:10.1002/cam4.71780)
Supplement: Supplementary file 1 — Data S1: Supporting Information. [file CAM4-15-e71780-s001.pdf]

科学研究项目伦理审查意见

（ 2023 ）年研审第（ 194）号

项目名称	PSAT1 介导的 SSP 通路与非小细胞肺癌的临床特征及预后的相关性		
项目类别	□国际多中心 □国内多中心 □列入各级各类科技计划项目<br>□ 横向课题 ■自主选题 □其他（病例报道）		
研究设计类别	■实验研究<br>□干预性研究<br>□观察性研究：□回顾性分析 □前瞻性研究 □现况观察性研究<br>利用人体组织和信息的研究：■以往采集保存 □研究采集		
组长单位			
申办单位	吉林大学第二医院		
申请科室	呼吸与危重症医学科	主要研究者	王珂
审查方式	□ 会议审查 ■ 快速审查		
送审材料	1. 研究方案（版本号：1.0，版本日期：2023.09.15）<br>2. 知情同意书（版本号：1.0，版本日期：2023.9.15）<br>3. 主要研究者履历		

审查意见：

根据国家卫生计生委《涉及人的生物医学研究伦理审查办法》(2016)、WMA《赫尔辛基宣言》(2013）和 CIOMS 联合 WHO《涉及人的健康相关研究国际伦理准则》（2016）的伦理原则，经本伦理委员会审查，意见如下：

同意

吉林大学第二医院医学伦理委员会

主任委员签字：杨薇印

2023 年 9 月 26 日

夸克扫描王
极速扫描，就是高效

附件 4

吉林大学动物实验伦理福利审查表

The Tab of Animal Experimental Ethical Inspection，JLU

编号(Number of permit)：SY202302013

<table>
<tr><td rowspan="9">申请人填写的相关信息
(Concerned information wrote by applicant)</td><td colspan="4">申请单位
(Name of organization)： 吉林大学第二医院</td></tr>
<tr><td colspan="4">申请人学历(Education of applicant) 博士　技术职称(Professional title)：教授　岗位证书编号(Certificate)：pzpx201812050408</td></tr>
<tr><td colspan="4">实验名称(Study title)：PSAT1 通过丝氨酸从头合成途径调控非小细胞肺癌的进展和药物治疗效果</td></tr>
<tr><td colspan="4">实验目的(Aim of experiment)：评估过表达及沉默 PSAT1 基因对肺癌细胞在裸鼠体内成瘤性。</td></tr>
<tr><td rowspan="4">拟进动物情况</td><td colspan="3">动物来源(Source of animal)：斯贝福（北京）生物技术有限公司</td></tr>
<tr><td colspan="3">品种品系(Species or strain)：Balb/c Nude mice 等级(Grade)：SPF 规格(Specifications)：4-6 week old</td></tr>
<tr><td>数量(Number)：♀ 24 只</td><td colspan="2">申请日期(Application date)：2022 年 11 月 6 日</td></tr>
<tr><td>进驻日期(Entering date)：2023 年 1 月 1 日</td><td colspan="2">结束日期(Ending date)：2023 年 3 月 30 日</td></tr>
<tr><td colspan="4">实验要点,包括实验方法、观测指标、实验结束后处死动物的方法等(Outline of experiments, experimental methods, observational index, executing animal method et. al)：
本实验在吉林大学实验动物中心屏障设施开展（SYXK（吉）2021-0006），在吉林大学动物伦理福利委员会监督下开展(IACUC)，遵守吉林大学及国家对于实验动物伦理福利的要求，饲养条件严格依照 GB14925 进行，实验动物自由饮水采食。
动物分组 4 组，分别空白组、对照质粒组、PSAT1 过表达组、PSAT1 沉默组。将肺癌细胞注入小鼠腋下皮下，建立裸鼠移植瘤模型。我们用电子卡尺测量肿瘤，接种 3-5 周后处死。
人道终点：肿瘤直径不得超过 1.5 ㎝。
安乐死方法：采用化学药物（吸入高浓度异氟烷）安乐死。
实验后，动物尸体集中无害化处理。</td></tr>
<tr><td></td><td colspan="4">申请人签名(Signature of applicant)：　联系电话(Telephone)：18643111766</td></tr>
<tr><td>审查依据
(Inspection contents)</td><td colspan="4">1. GB/T 35892-2018 实验动物 福利伦理审查指南 (Laboratory Animal – Guideline for ethical review of animal welfare)。
2. 实验动物饲养管理和使用指南，第 8 版，上海科技出版社（Guide for the Care and Use of Laboratory Animals: Eighth Edition）。</td></tr>
<tr><td rowspan="2">审查结果
(是否同意申请人的实验方案)
(Results of inspection)</td><td>委员会专家意见
(Study director)：</td><td>同意
(Agree) ✓</td><td>不同意
(Disagree) □</td><td>签 名
(Signature)：</td></tr>
<tr><td>吉林大学实验动物福利伦理委员会
(Institutional Animal Care and Use Committee of Jilin University，IACUC)：</td><td>同意
(Agree) ✓</td><td>不同意
(Disagree) □</td><td rowspan="2">签章(Stamp)
吉林大学实验动物中心
伦理审查专用章</td></tr>
<tr><td colspan="4">备注(Supplement)：</td></tr>
</table>

夸克扫描王
极速扫描，就是高效
