# Supplementary material for: PSAT1 Promotes NSCLC Progression via the De Novo Serine Synthesis Pathway and Represents a Therapeutic Vulnerability
Source: Cancer Med. 2026 Apr 2;15(4):e71780. doi: 10.1002/cam4.71780 (PMC13045451; doi:10.1002/cam4.71780)

Figure S1 Scoring standards of IHC staining

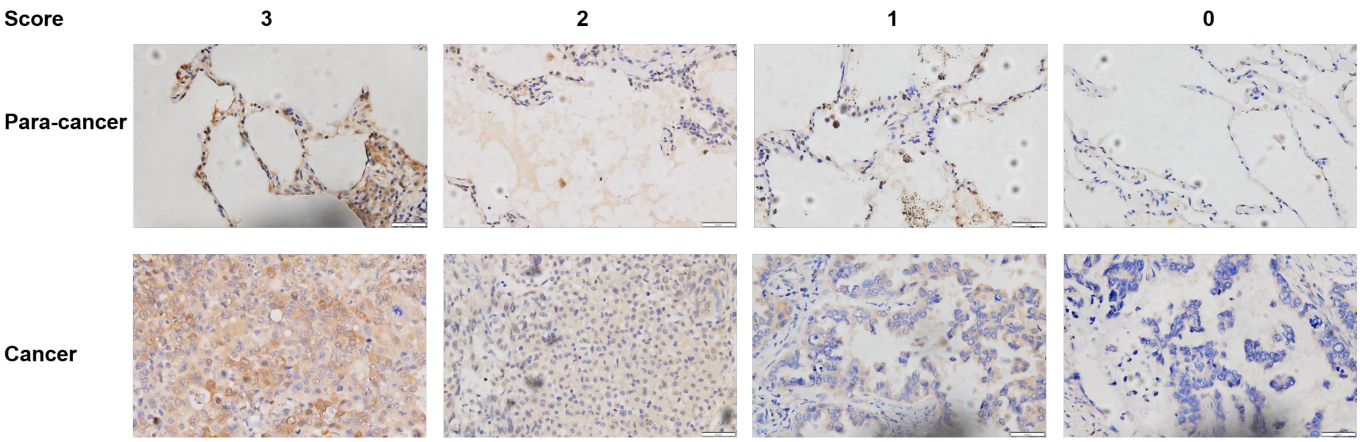

Figure S2 CCK-8 assay conducted on H1299 cell line transfected with shB and shC

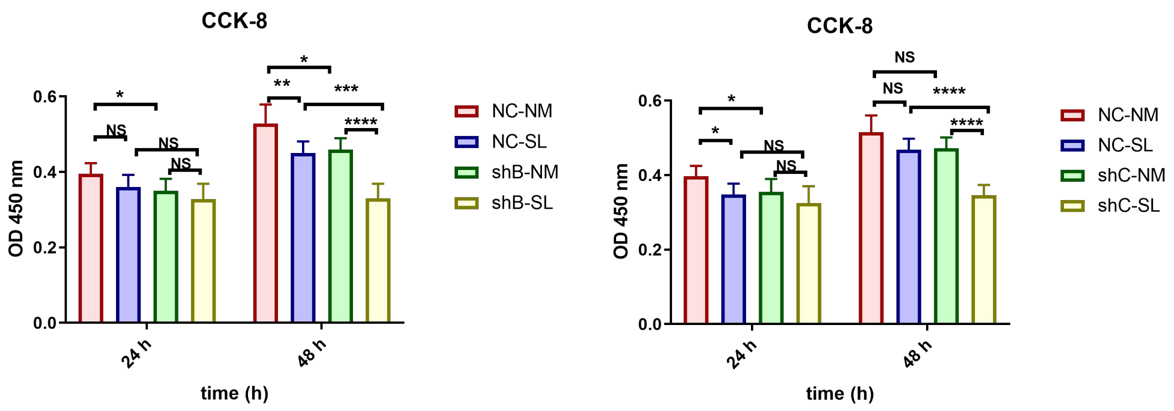

Figure S3 Original figures of ROS

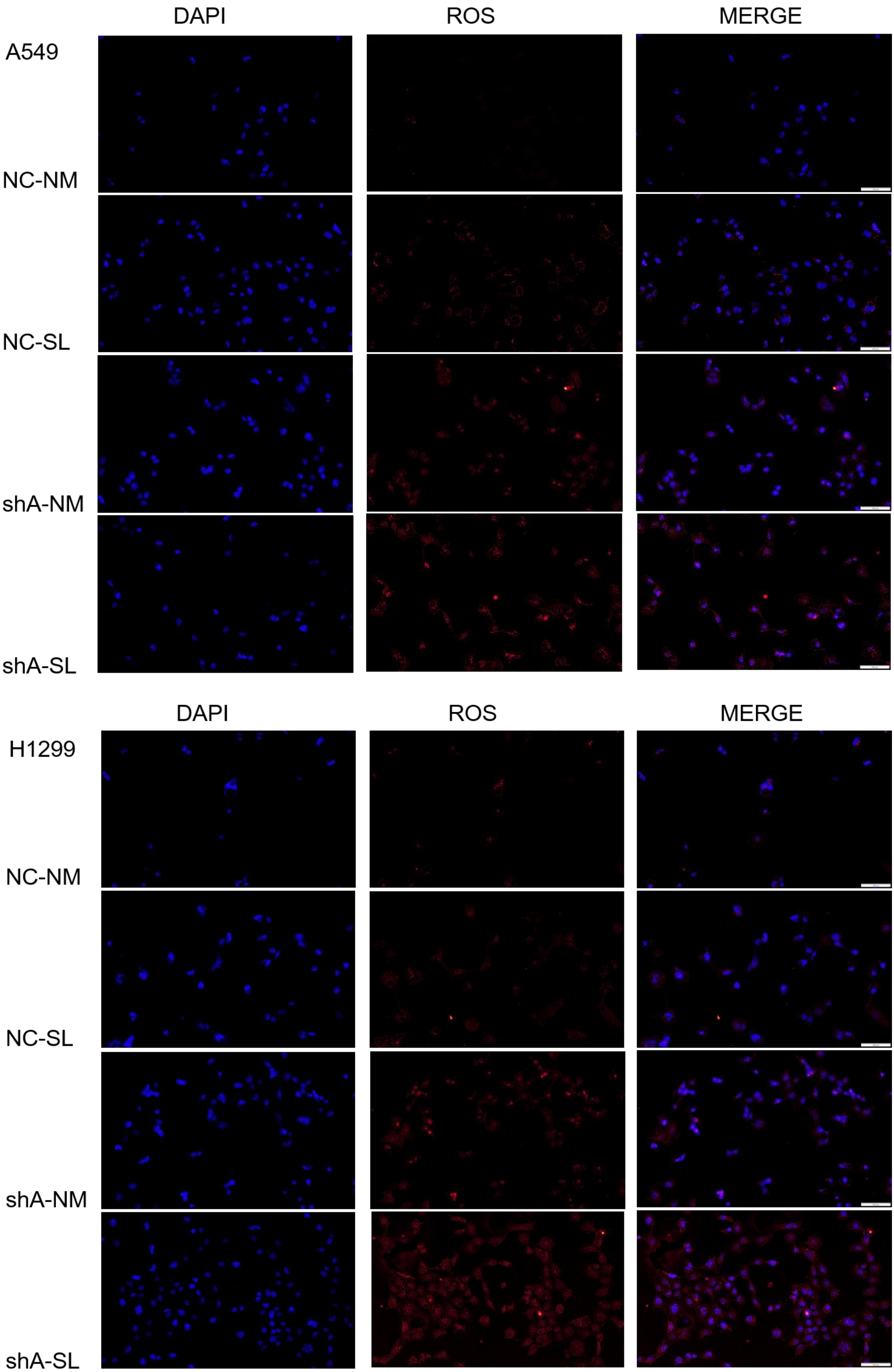

Figure S4 Original figures of ROS (adding ROS scavengers NAC)

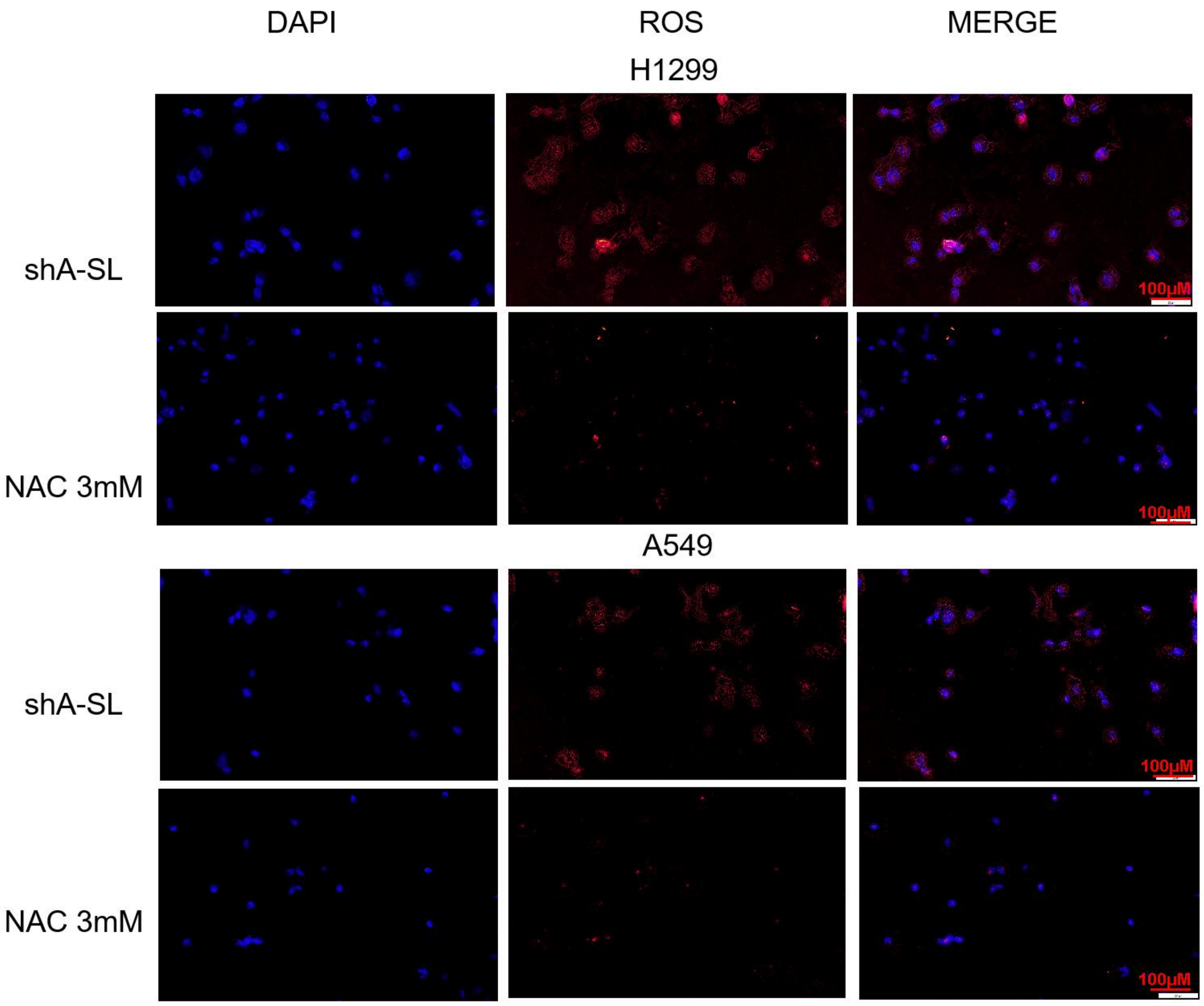

Figure S5 Inhibition of NF-κB rescues apoptosis and attenuates ROS elevation induced by PSAT1 knockdown under serine deprivation.

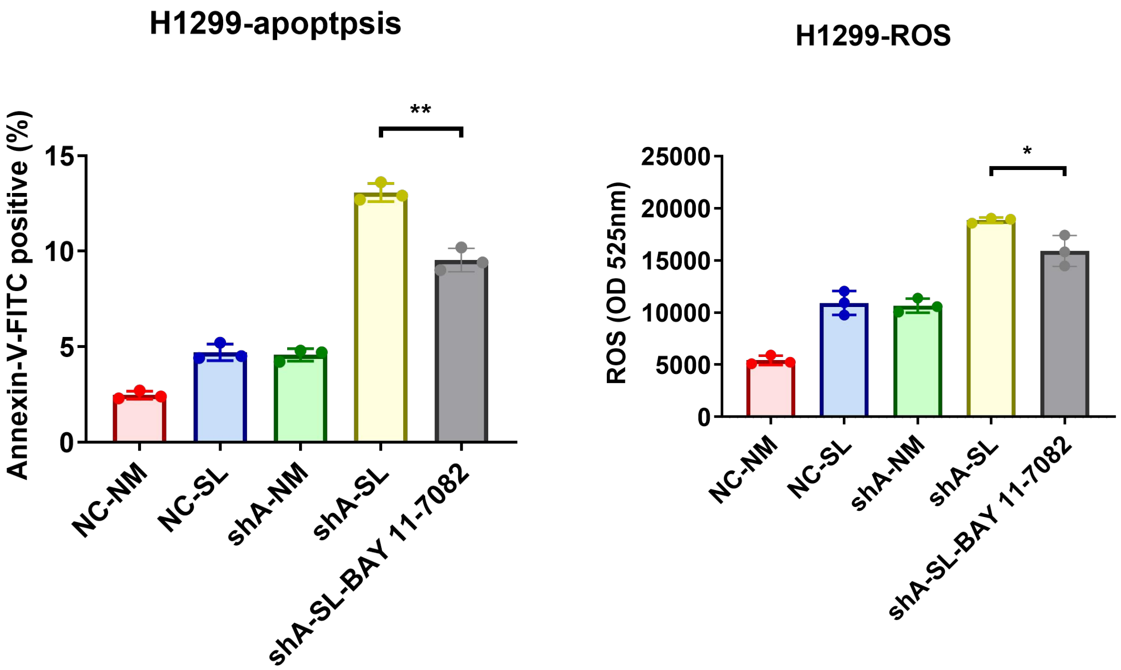

Figure S6 Flow cytometric analysis of cleaved caspase-3 in H1299 cells

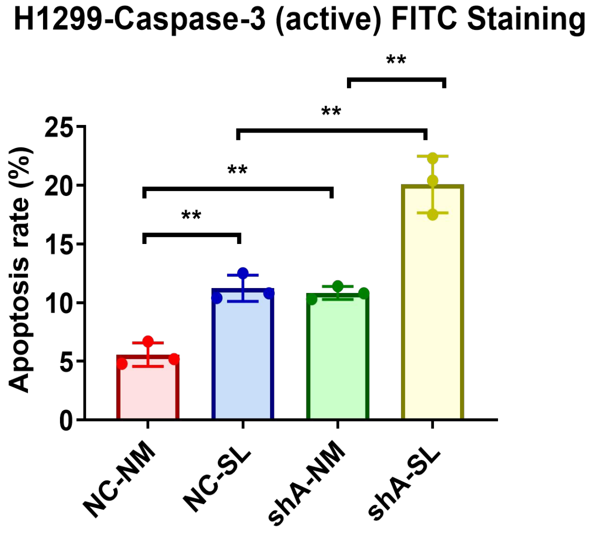

Figure S7 All map of isotop labeling experiment

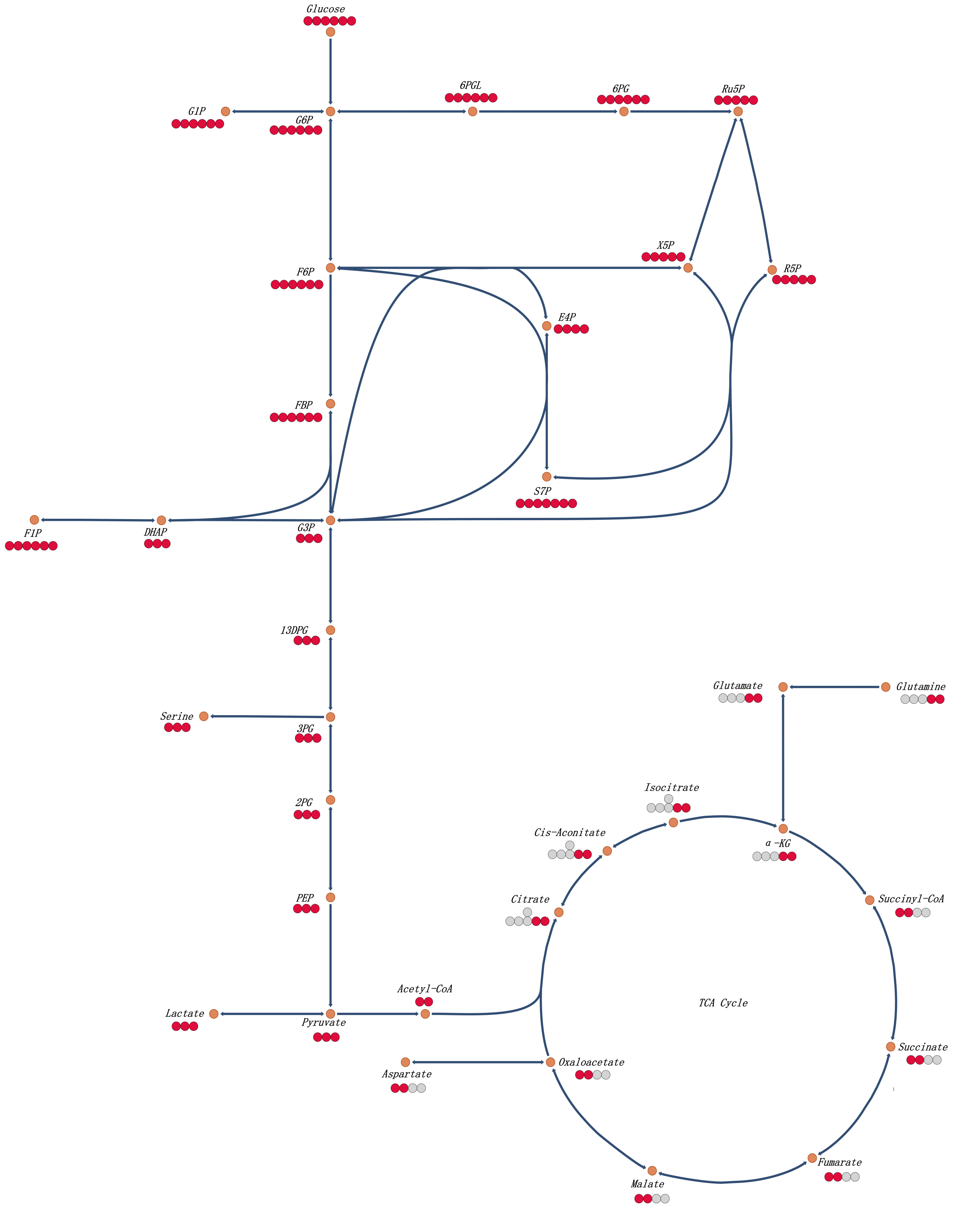

Figure S8 Isotope labeling experiment using A549 cell line

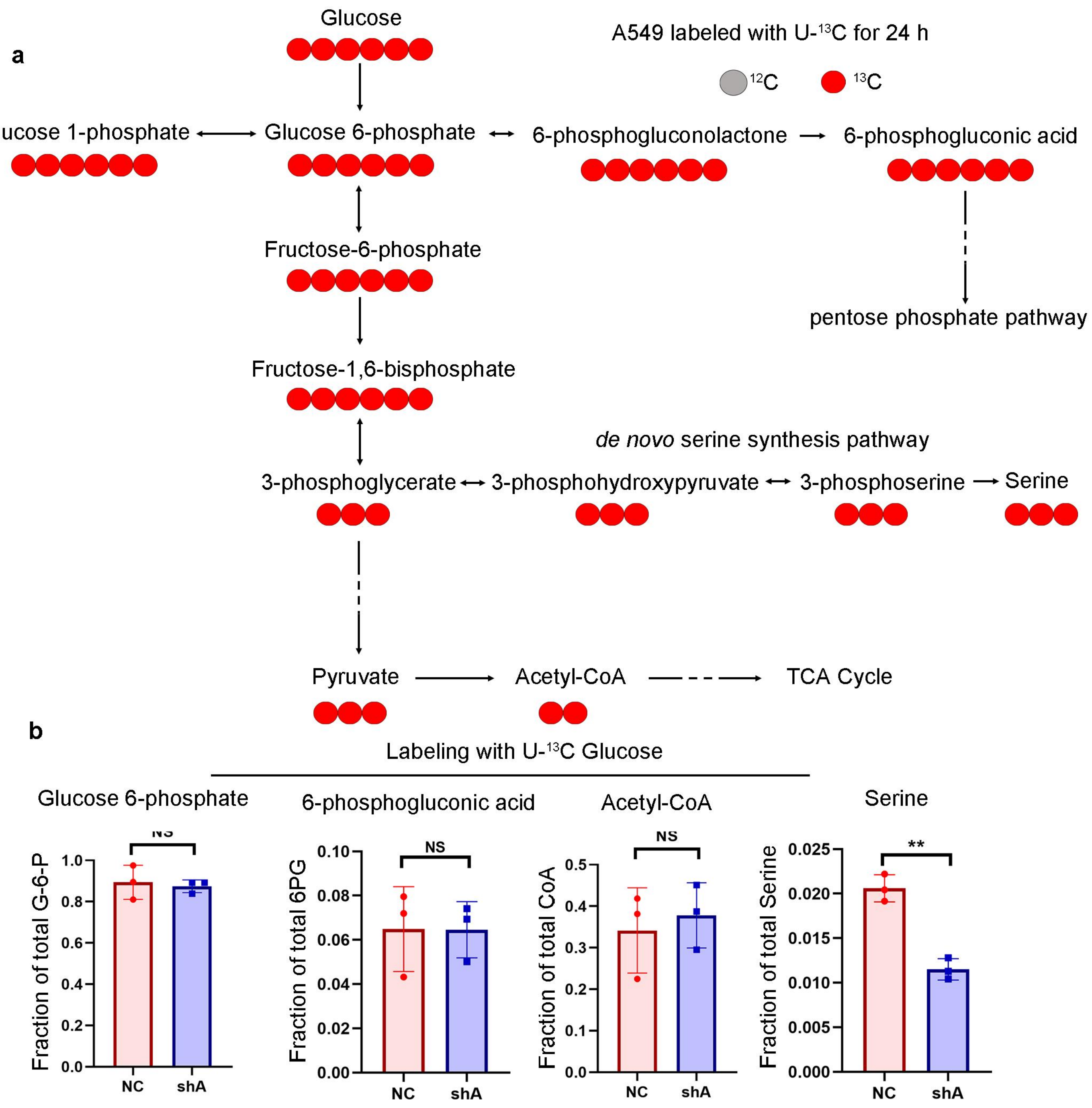

Supplement: Supplementary file 2 — Figure S1: Scoring standards of IHC staining. Figure S2: CCK‐8 assay conducted on the H1299 cell line transfected with shB and shC. Figure S3: Original figures of ROS. Figure S4: Original figures of ROS (adding ROS scavengers NAC). Figure S5: Inhibition of NF‐κB rescues apoptosis and attenuates ROS elevation induced by PSAT1 knockdown under serine deprivation. Figure S6: Flow cytometric analysis of cleaved caspase‐3 in H1299 cells. Figure S7: All maps of the isotope labeling experiment. Figure S8: Isotope labeling experiment using the A549 cell line. [file CAM4-15-e71780-s002.pdf]
